# PVA Nanofibers by Solution Blow Spinning: Processing Principles, Challenges, and Biomedical Applications

**DOI:** 10.3390/polym18182253

**Published:** 2026-09-16

**Authors:** Fazila Ashraf, Dania Olmos, Javier González-Benito

**Affiliations:** Materials Science and Engineering and Chemical Engineering, (IAAB) Universidad Carlos III de Madrid (UC3M), 28915 Leganes, Madrid, Spain; fashraf@ing.uc3m.es

**Keywords:** solution blow spinning (SBS), polyvinyl alcohol (PVA), nanofibers, biomedical applications, nanoparticles, hydrogels

## Abstract

Poly(vinyl alcohol) (PVA) nanofibers have emerged as versatile materials in biomedical science due to the high surface-to-volume ratio enabled by nanofibrous morphologies, together with their biocompatibility, hydrophilicity, low toxicity, water solubility, and ease of chemical modification. Solution blow spinning (SBS) provides an alternative route for producing PVA-based nanofibers and is particularly relevant for PVA because the polymer is commonly processed from aqueous solutions. This review critically examines the fabrication of PVA nanofibers by SBS within the broader framework established for PVA hydrogels and electrospun PVA nanofibers, emphasizing how aqueous processing conditions govern fiber formation and morphology and how these features translate into mechanical performance and functional behavior tailored to biomedical applications. Applications in wound dressings, drug-delivery systems, tissue engineering scaffolds, and biosensors are discussed, highlighting the role of the fillers/additives in modulating biological responses and drug release behavior. Key challenges remain, including water sensitivity and PVA solubility, crosslinking approaches compatible with bioactive payloads, and limitations in mechanical robustness for certain load-bearing applications. Finally, future perspectives on scale-up and eco-friendly PVA nanofibers are outlined to support translation of SBS-derived PVA nanofibers toward clinical applications. Overall, this work positions SBS as a promising route to complement established nanofiber fabrication methods and support the sustainable integration of PVA nanofibers into next-generation biomedical solutions, while providing broader insights into the processing of aqueous polymer systems by SBS.

## 1. Introduction

Over the past few decades, polymer nanofibers have attracted the attention of researchers and industries alike, primarily due to their remarkable properties, such as a high surface-to-volume ratio, porosity control, and stability across an extensive range of applications [1,2]. Owing to their fibrous microstructure, polymer nanofibers closely resemble the extracellular matrix (ECM) [3], which promotes cell adhesion, proliferation, and differentiation, thereby supporting tissue regeneration and repair. Furthermore, by incorporating functional nanoparticles into polymer nanofibers, it is possible to expand their applicability by enabling tailored mechanical, biological, electrical, and antimicrobial properties. Such functionalization strategies [4,5] have facilitated the development of advanced wound dressings [6], targeted drug-delivery systems, and tissue engineering scaffolds designed to enhance healing outcomes [7] and minimize adverse effects [8,9]. In addition, nanofiber-based materials have demonstrated potential in implantable medical devices, where biocompatibility and structural adaptability are essential [10].

Among the many polymers, poly(vinyl alcohol) (PVA) stands out because of its biocompatibility, biodegradability, water solubility, and processability [11,12,13]. Its non-toxic nature and structural resemblance to the extracellular matrix when it is prepared in the form of fibers make PVA especially promising for biomedical applications, as it provides an environment that closely resembles the natural tissue [14]. Extensive research has been conducted on electrospun PVA nanofibers [15,16,17,18], which have great potential for applications such as controlled drug release and the design of advanced scaffolds for tissue engineering [14,19]. By leveraging nanotechnology through nanofiber-based materials, there is a significant opportunity to enhance patient health, expedite recovery, and promote effective healing [20]. These unique attributes of PVA nanofibers can transform the biomedical field by offering innovative solutions to persistent challenges in modern medicine. These characteristics have established PVA as one of the most extensively investigated polymers for biomedical nanofibers, providing a solid scientific foundation for understanding the relationships between polymer composition, processing, structure, and biological performance.

Extensive research on PVA (polyvinyl alcohol) hydrogels [21] and electrospun nanofibers [22,23] has demonstrated the biomedical potential of PVA and provided a thorough understanding of its structure-property relationships. In contrast, the application of solution blow spinning (SBS) to PVA remains relatively underexplored, even though there is growing interest in this technique as a complementary method for fabricating nanofibers. Unlike many polymers that are commonly processed by SBS using volatile organic solvents, PVA is typically processed from aqueous solutions. This introduces additional challenges related to solvent evaporation, jet stability, and fiber formation. As a result, PVA presents a unique opportunity to investigate not only the biomedical potential of nanofibers made using SBS but also the broader scientific principles governing the processing of aqueous polymer solutions with this technique.

Considering this background, this review critically examines the development of PVA nanofibers fabricated by solution blow spinning (SBS), with particular emphasis on biomedical applications. By integrating the knowledge established through PVA hydrogels and electrospun nanofibers with the emerging SBS literature, the review discusses how solution blow spinning expands the possibilities for fabricating PVA-based nanomaterials while highlighting the distinctive challenges associated with processing aqueous polymer systems. In addition, the current limitations of the field and future research directions are critically discussed to provide a comprehensive perspective on the development of SBS-fabricated PVA nanofibers.

**Figure 1:**
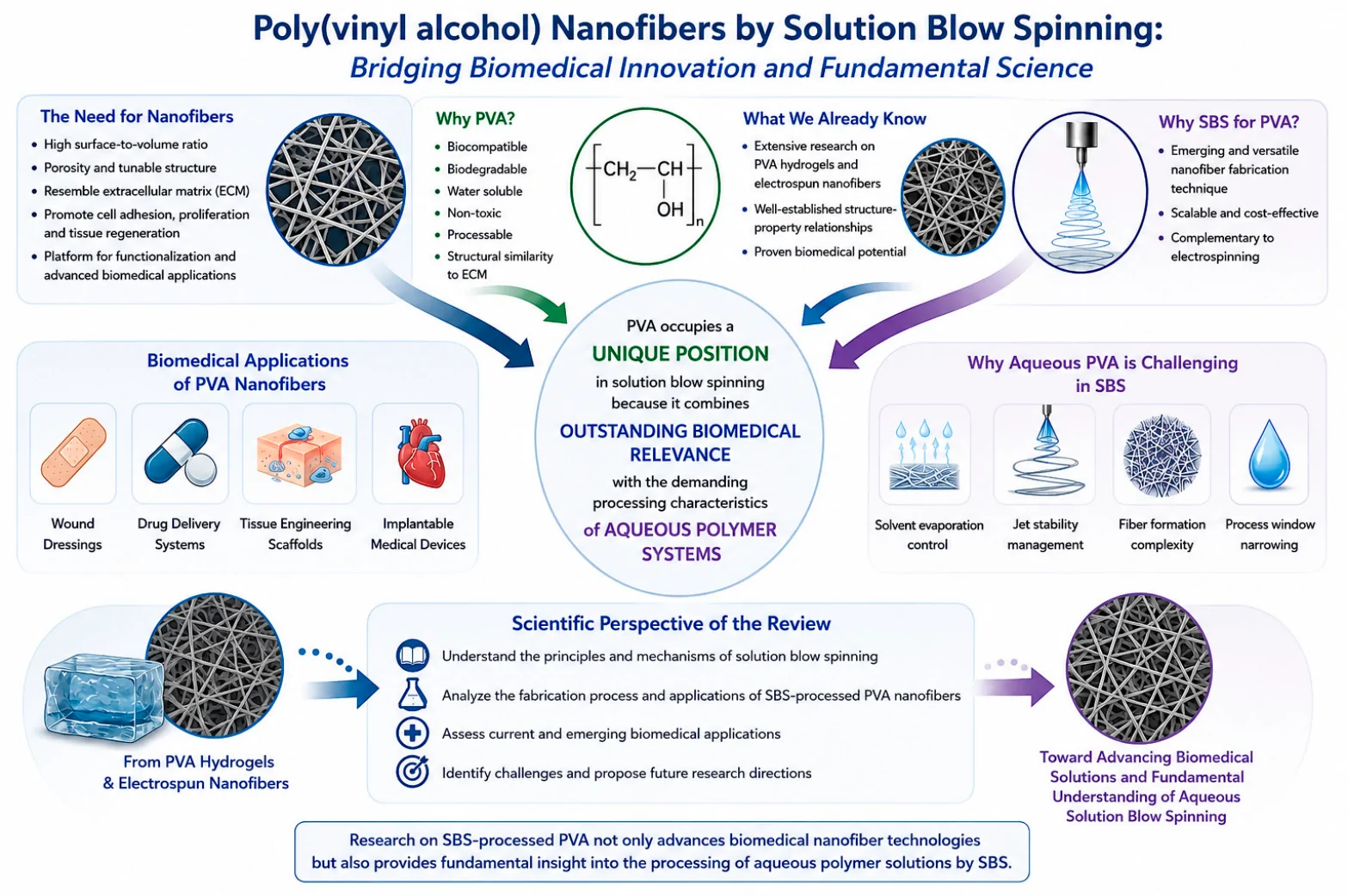
Conceptual framework of the review, illustrating the unique role of poly(vinyl alcohol) (PVA) in solution blow spinning (SBS). The scheme links the established knowledge on PVA hydrogels and electrospun nanofibers with the emerging opportunities and challenges of SBS-fabricated PVA nanofibers for biomedical applications.

The scope and scientific perspective of this review are summarized schematically in Figure 1. Rather than considering PVA solely as a biomedical polymer, the figure highlights its unique position within solution blow spinning, where its well-established biomedical relevance converges with the distinctive challenges associated with processing aqueous polymer solutions. This dual perspective provides the conceptual framework that underpins the discussion presented throughout the review, linking the extensive knowledge derived from PVA hydrogels and electrospun nanofibers with the emerging advances in SBS-fabricated PVA nanofibers.

## 2. Solution Blow Spinning: Fundamentals and Processing Principles

Several techniques have been developed for the fabrication of polymer nanofibers, including electrospinning (ES), melt spinning (MS), centrifugal spinning (CS), and, more recently, solution blow spinning (SBS). Although all these methods produce fibrous materials, they rely on different fiber formation mechanisms and processing conditions, making each technique suitable for specific polymers and applications [24,25].

These techniques enable the production of fibers with diameters ranging from tens of nanometers to several micrometers, thereby supporting a wide range of applications. The versatility of electrospinning (ES), solution blow spinning (SBS), and melt spinning (MS) lies in their ability to tailor fiber morphology, porosity, and diameter [26]. This tunability can be achieved by adjusting and optimizing process parameters such as polymer concentration, temperature, humidity, gas or electric field strength, and collector design. Ongoing advancements in nanofiber processing are rapidly expanding the range of processable materials and facilitating the transition from laboratory-scale to industrial production, successfully bridging the gap. This includes the use of biopolymers such as chitosan [27,28], gelatin [29], and zein [30,31], inorganic composites (like hydroxyapatite) [32], alginate [33], and carbon quantum dot (CQD)-doped titanium dioxide (TiO_2_) [34], and sophisticated nanoparticle-loaded blends containing functional additives such as metal oxides, ceramics, graphene oxide, and antibacterial agents. These advancements in material properties, coupled with improved throughput and scalability, are essential for driving nanofiber technology toward widespread industrial application [35,36].

Melt spinning (MS) involves extruding a polymer melt through a spinneret, followed by drawing and solidification of the molten filament. By eliminating solvents, MS delivers environmental benefits, and high throughput is suitable for industrial-scale production; however, only thermally stable polymers that can tolerate processing temperatures typically above 200 °C are compatible. The resulting fiber diameters generally exceed one micrometer, which may limit applicability in biomedical contexts requiring nanoscale fibers. Operationally, melt spinning requires extrusion and subsequent drawing of polymer strands, but this process is also restricted to materials capable of tolerating the mechanical and shear stresses without degradation [37,38].

Centrifugal spinning (CS) uses centrifugal force and air drag to attenuate polymer melts or solutions into fibrous forms. The primary advantage of CS lies in its significantly higher production rate and throughput compared to traditional methods like electrospinning. While the process eliminates the need for high-voltage power supplies, it typically produces thicker, unaligned fibers. Furthermore, achieving precise fiber alignment remains a challenge, and the high-speed rotational components require robust safety shielding during operation [39].

Electrospinning (ES) employs a high-voltage electric field (typically 14–40 kV) to draw a polymer solution. Polymer solutions with concentrations commonly between 5 and 30 wt% are delivered through a fine capillary when electrostatic repulsion exceeds surface tension. Continuous nanofibers are formed when the solvent is conveniently evaporated and collected on a grounded target [5,40,41]. The morphological and mechanical characteristics of fine nanofibers can be adjusted by changing the solution concentration, solution flow rate, voltage, working distance between the needle and collector, needle diameter, and also by utilizing a controlled system of humidity and temperature during the process [41,42]. Despite its ability to produce highly uniform nanofibers, electrospinning is constrained by relatively low production rates and safety considerations associated with high-voltage operation. Usually, a lower production rate is addressed by the use of multiple needle setups. With a focus on innovation, the potential uses of this technology are broad and promising.

**Figure 2:**
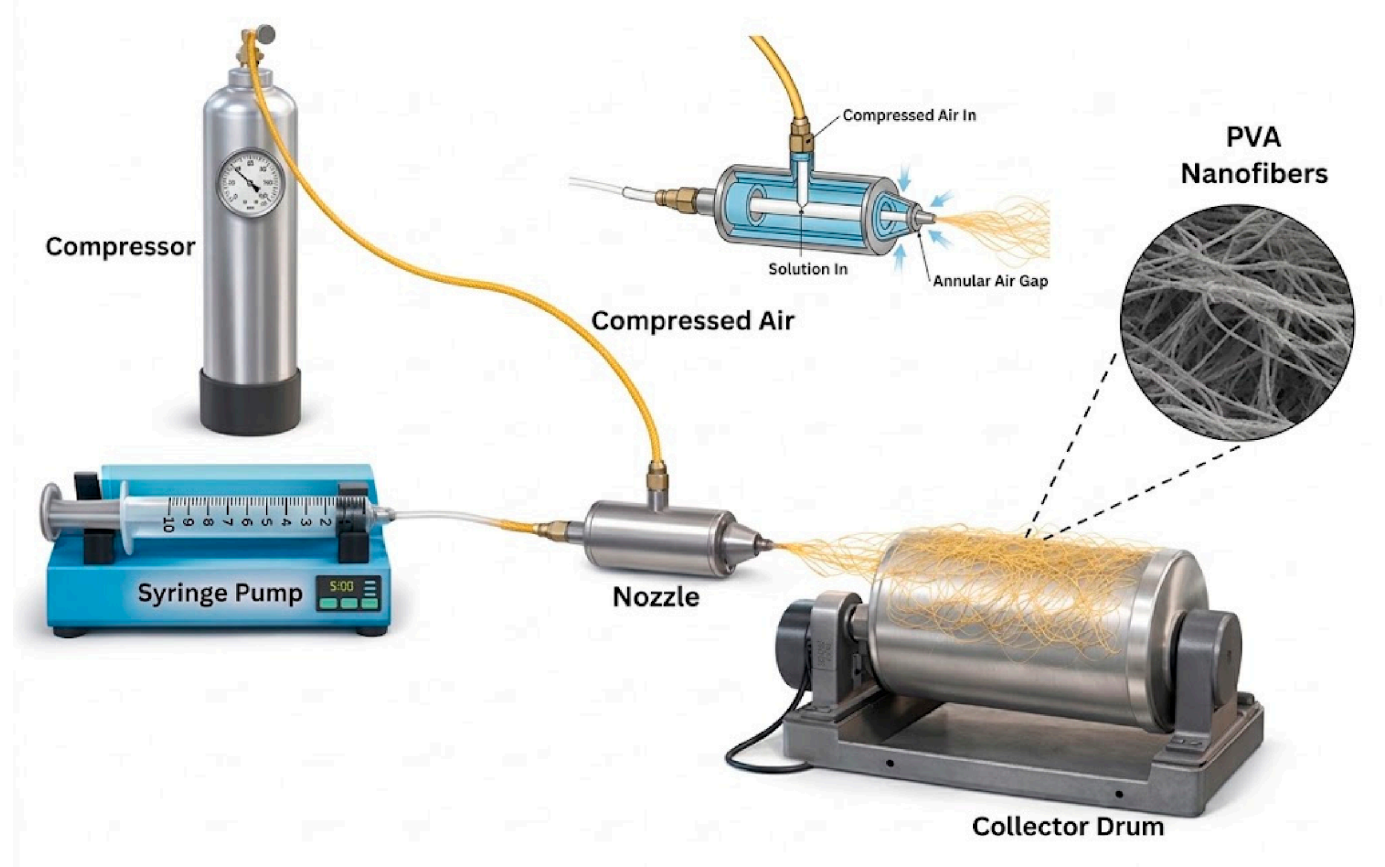
Schematic of the solution blow spinning (SBS) process.

Solution blow spinning (SBS) is an innovative fiber production technique that utilizes high-velocity gas to draw a polymer solution through a nozzle, eliminating the need for electric fields. In contrast, electrospinning relies on electrostatic forces to produce highly uniform nanofibers but is often limited by relatively low production rates and safety concerns associated with high-voltage operation, which hinder large-scale industrial implementation [5,40,41,42,43]. As illustrated schematically in Figure 2, solution blow spinning (SBS maintains a balance by enabling ultrafine fiber formation through aerodynamic forces, with fiber diameters that can be finely controlled by adjusting solution viscosity, flow rate, and process parameters. It operates effectively without high voltage or thermal constraints, making it suitable for a broad range of applications. SBS process uses compressed gas to stretch a polymer solution through a concentric nozzle, with aerodynamic forces drawing the jet as the solvent evaporates and fibers are deposited on a collector. This combination of high throughput, safety, and versatility positions SBS as a promising technology for scalable fiber manufacturing. Owing to its high productivity, operational simplicity, and broad compatibility with polymer solutions, SBS has emerged as a promising alternative for the scalable production of polymeric micro- and nanofibers.

Table 1 provides a detailed comparison of the fundamental characteristics of these techniques, including their working principles, driving forces, feed materials, and solvent requirements. Although all four techniques can produce fibrous materials, they rely on different fiber formation mechanisms, resulting in distinct processing capabilities and application domains.

Among these processing techniques, solution blow spinning (SBS) has attracted increasing attention as a versatile nanofiber fabrication technique based on the aerodynamic stretching of polymer solutions by a high-velocity gas stream [4]. Consequently, SBS offers broad flexibility in terms of material selection, solvent systems, and operating conditions, while also enabling relatively high production rates, making SBS an attractive approach for scalable nanofiber manufacturing for various applications, including biomedical ones [44,45].

The characteristics of the fibers produced by each processing technique together with their principal processing parameters are summarized in Table 2. In SBS, fiber morphology and quality are governed by the combined effects of gas pressure, polymer flow rate, working distance, nozzle geometry, solution properties, and environmental conditions such as temperature and relative humidity. Careful optimization of these parameters is required to obtain defect-free fibers with the desired morphology and functional performance.

**Table 1:** Fundamental characteristics of nanofiber fabrication techniques [26,36,39,46,47].

Feature	Solution Blow Spinning (SBS)	Electrospinning (ES)	Melt Spinning (MS)	Centrifugal Spinning (CS)
Working Principle	Uses compressed gas to eject polymer solution and form fibers	Uses a high-voltage electric field to draw charged polymer solution into fibers	Uses heat to melt polymer, extrudes, and solidifies fibers	Uses centrifugal force and air drag to spin the polymer through the rotating nozzle
Driving Force	Compressed gas/air	Electrostatic force	Mechanical extrusion	Centrifugal force
Feeding Material	Polymer solution	Polymer solution or emulsion	Polymer melt	Polymer melt or solution
Solvent Required	Yes	Yes	No	Optional

**Table 2:** Fiber characteristics and processing parameters.

Feature	Solution Blow Spinning (SBS)	Electrospinning (ES)	Melt Spinning (MS)	Centrifugal Spinning (CS)
Fiber Diameter	~200–800 nm [46,48]	~100–500 nm [36,46]	>1 µm [47]	100–2000 nm [39]
Fiber Morphology	Continuous fibers with broader diameter distribution; tunable morphology via processing parameters	Uniform, smooth fibers, narrow diameter distribution; highly tunable	Smooth, continuous fibers with relatively large diameters	Aligned or random, depending on the speed of the centrifuge
Key Control Parameters	Air pressure, nozzle geometry, flow rate, and working distance	Voltage, working distance, flow rate	Temperature, screw speed, die design	Rotational speed, nozzle diameter, solution viscosity, temperature

As illustrated in Table 1 and Table 2, each processing technique possesses inherent advantages and limitations arising from its underlying fiber formation mechanism. Melt spinning is well suited for thermoplastic polymers but is restricted by the requirement for thermal processability. Centrifugal spinning offers high productivity but generally produces fibers with broader diameter distributions, whereas electrospinning enables excellent control over fiber morphology at the expense of relatively low production rates. In contrast, solution blow spinning combines relatively high productivity with simple equipment and broad material compatibility, explaining its growing interest as a complementary manufacturing technique for polymeric nanofibers. The fundamental principles governing SBS and the influence of the main processing parameters on fiber formation are discussed in the following sections, with particular emphasis on the processing of aqueous PVA solutions.

The mechanism of SBS is governed by the interaction between the high-speed gas stream and the polymer solution jet. According to Bernoulli’s principle, the high gas velocity surrounding the polymer droplet generates a pressure differential that accelerates the solution and promotes jet formation. When a droplet of polymer solution is subjected to the higher pressure at the nozzle and the high gas velocity, it triggers a pressure drop at the center of the jet. This drop creates a driving force that accelerates the polymer solution. The droplet transforms into a conical shape, and as the high-velocity air exerts a shearing force that exceeds the surface tension of the polymer solution, a polymer jet is formed and propelled toward the collector. Pressurized air helps to evaporate the solvent, and the polymer fibers are finally collected on the collector at a certain distance from the nozzle [49].

**Figure 3:**
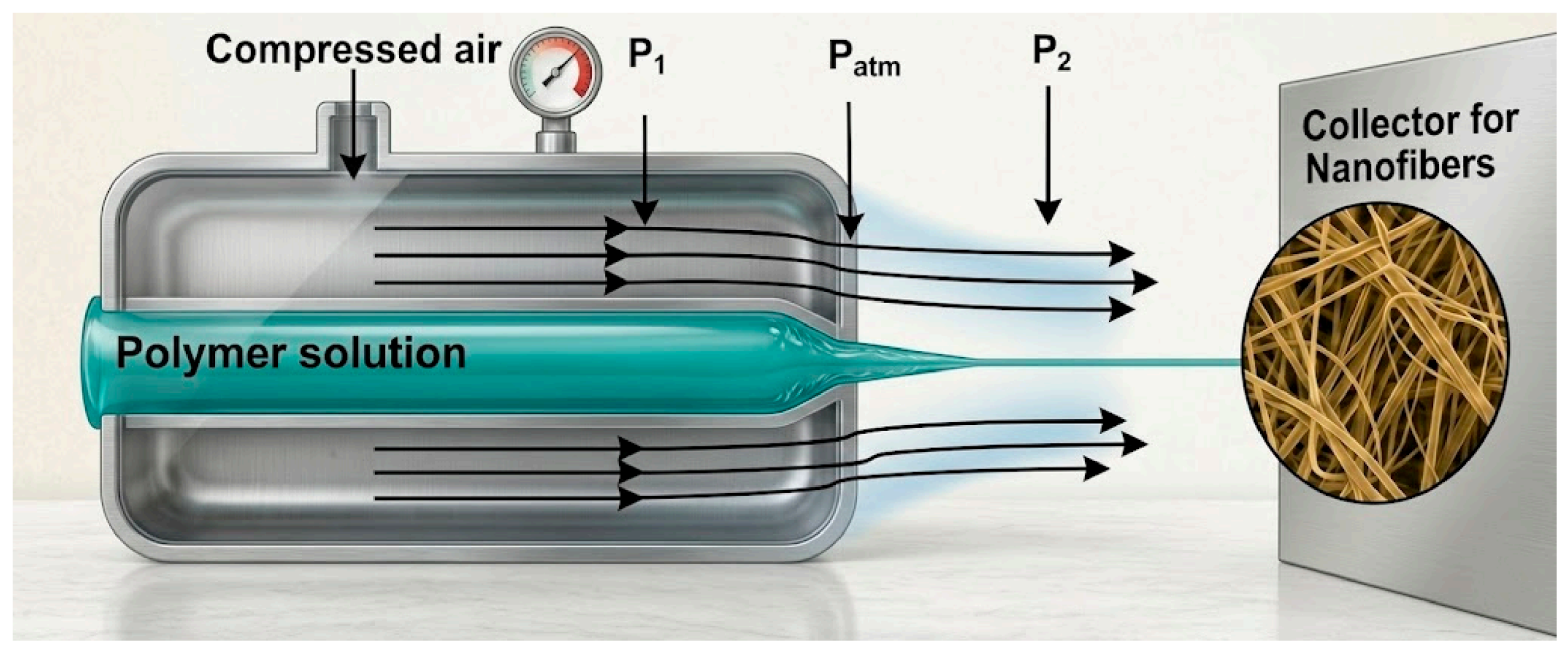
Schematic of a concentric nozzle for SBS.

The interaction between the gas stream and the polymer solution is crucial in determining the morphology and characteristics of the resulting nanofibers [45]. As illustrated in Figure 3, the working mechanism of SBS involves the jet encountering different pressures P_1_, P_atm_, and P_2,_ depending on its position. As P_atm_ is lower than P_1_, it naturally drives the jet towards the collector, highlighting the influence of pressure differences in achieving the fibers [36].

**Figure 4:**
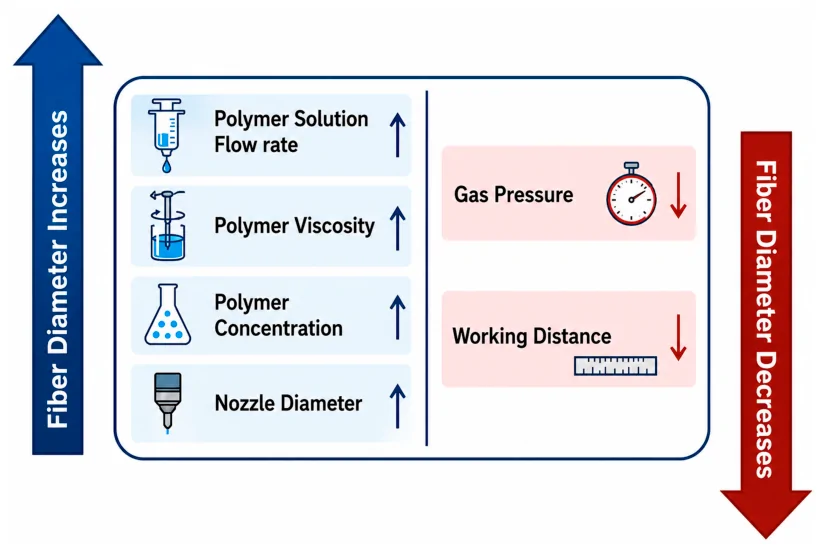
Scheme illustrating factors affecting the fiber diameter.

The morphology and structural characteristics of SBS-fabricated nanofibers (fiber diameter, orientation, and alignment) are strongly influenced by the interplay between gas dynamics and polymer solution properties. By carefully controlling these parameters, it is possible to precisely tailor nanofiber characteristics to meet specific application requirements. Employing highly volatile solvents can significantly accelerate fiber formation [4], yet achieving optimal fiber quality depends on maintaining a delicate balance among viscosity, surface tension, and evaporation rate to prevent nozzle clogging and ensure consistent fiber production. The solution and the experimental equipment determine the final nanofiber properties. To prevent bead formation and fiber entanglement in a solution, concentration and viscosity are related and can be controlled by adjusting the polymer concentration. It has been observed that polymer concentration is the most crucial factor in achieving homogeneous, continuous nanofibers without clusters or beads [50,51]. It determines solution viscosity and chain entanglement and should be maintained in an optimal range. If the concentration is too low, chains will not tangle, whereas if it is too high, the solution will become too viscous to work with. It is recommended that polymer concentration should not be below a certain critical concentration, c*, which is called the overlap concentration [52], a limit between diluted and semi-diluted concentrations [53]. It is often cited that the critical entanglement concentration (c_e_) should be 10 times the overlap concentration. Below this concentration, polymer chains remain unentangled and have lower internal cohesion to stabilize the fiber jet. Therefore, it is necessary to process the solution above the overlap concentration to attain a continuous fiber rather than fragmented beads [51,54]. Fiber morphology is defined by the interplay of surface tension and gas pressure, which collectively create the necessary shear forces at the air–liquid interface (Figure 4). The key factors influencing fiber diameter and morphology are summarized in Figure 4, highlighting the importance of precise parameter control for successful nanofiber fabrication.

**Figure 5:**
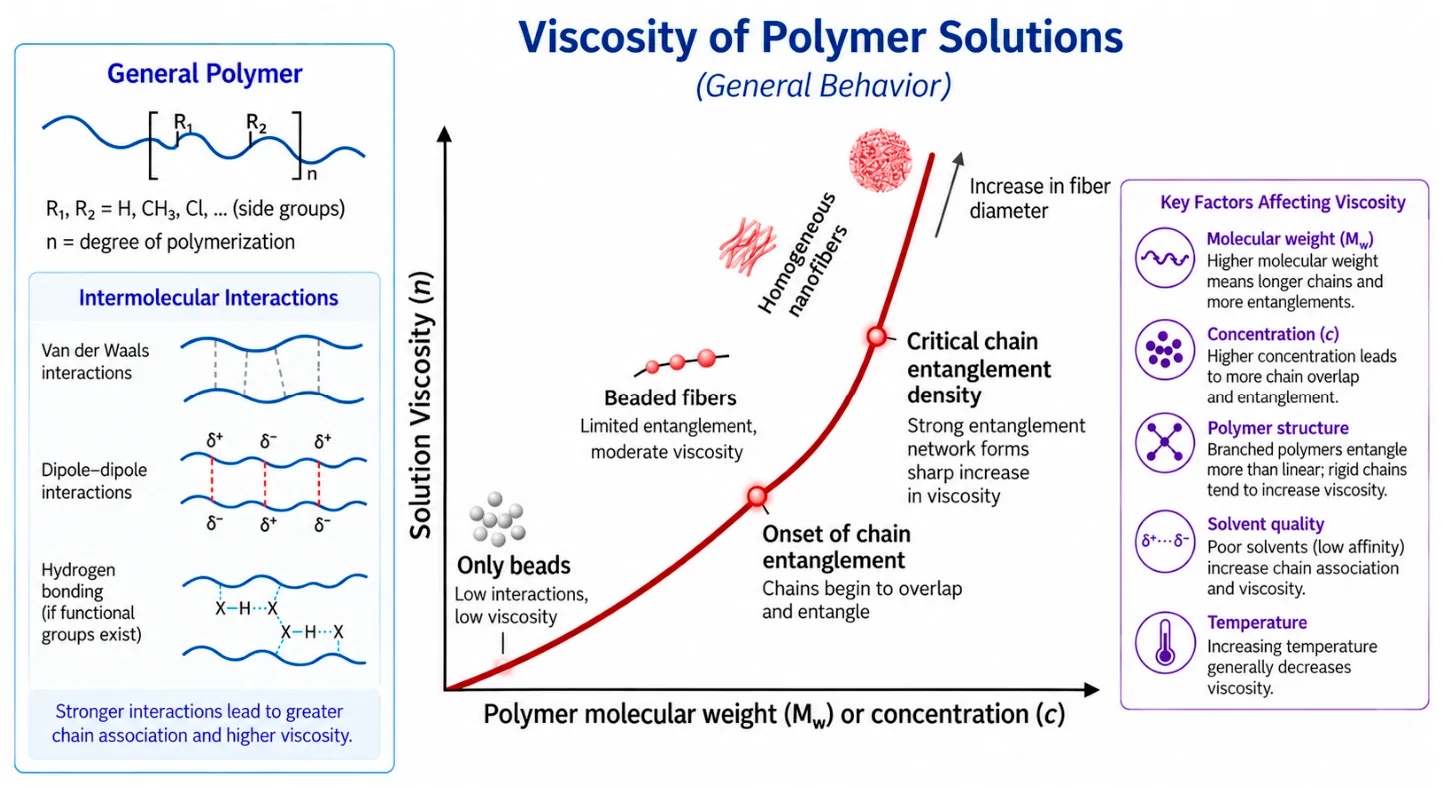
Factors governing the viscosity of polymer solutions. Effect of polymer molecular weight and concentration on the formation of polymer nanofibers.

Determining the optimal gas pressure is a critical and complex process because the actual force exerted on the polymer solution depends entirely on the nozzle’s physical characteristics (size and shape). Therefore, the nozzle’s design is the key limiting factor for effective pressure [55]. Also, it is observed that homogeneous fibers can be obtained by increasing the distance between the needle tip and the collector, as it can give sufficient time for the solvent to evaporate from the nanofibers before reaching the collector [5,56]. The tangential speed of the collector provides the necessary aerodynamic drawing forces to stretch the nanofibers and guide their deposition. The high-speed rotating collector facilitates fiber alignment and orientation [57,58]. Similarly, higher concentration and molecular weight can assist in attaining a stable fiber jet (Figure 5), but beyond a certain limit (which varies from polymer to polymer), they may clog the nozzle and result in nonuniform fibers and material spitting [55,57,58]. The feeding rate should be maintained after several trials, as a high feeding rate can destabilize the fiber jet, whereas a lower feeding rate can lead to curled or discontinuous fiber morphology [5].

## 3. PVA in Biomedical Applications: From Hydrogels to Nanofibers

PVA has been extensively investigated as a biomaterial in two principal forms: hydrogels and electrospun nanofibers. The work carried out in both areas establishes the physicochemical and biological properties that make PVA attractive for biomedical use, and serves as the reference framework against which SBS-derived PVA nanofibers can be evaluated. The following sections review this literature with that purpose, identifying which functionalities are well established and which remain to be demonstrated when PVA is processed by SBS.

**Figure 6:**
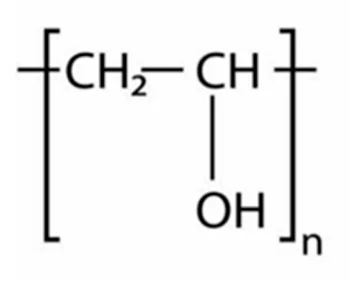
Chemical structure of polyvinyl alcohol (PVA).

As discussed, polyvinyl alcohol (PVA) is a hydrophilic synthetic polymer widely investigated for biomedical applications due to its biocompatibility, non-toxicity, and ability to be blended or functionalized to enhance its mechanical and surface properties. PVA cannot be directly synthesized by the polymerization of vinyl alcohol monomer due to its inherent instability. Instead, PVA is produced through the hydrolysis of polyvinyl acetate and is commonly used as a water-soluble plastic in many commercial applications [59,60]. PVA, being a synthetic material, is biodegradable, and its degradability is defined and enhanced through hydrolysis due to the presence of hydroxyl groups (Figure 6) on the hydrocarbon chain, as well as by the tacticity of PVA, which ultimately defines the final characteristics [61].

**Figure 7:**
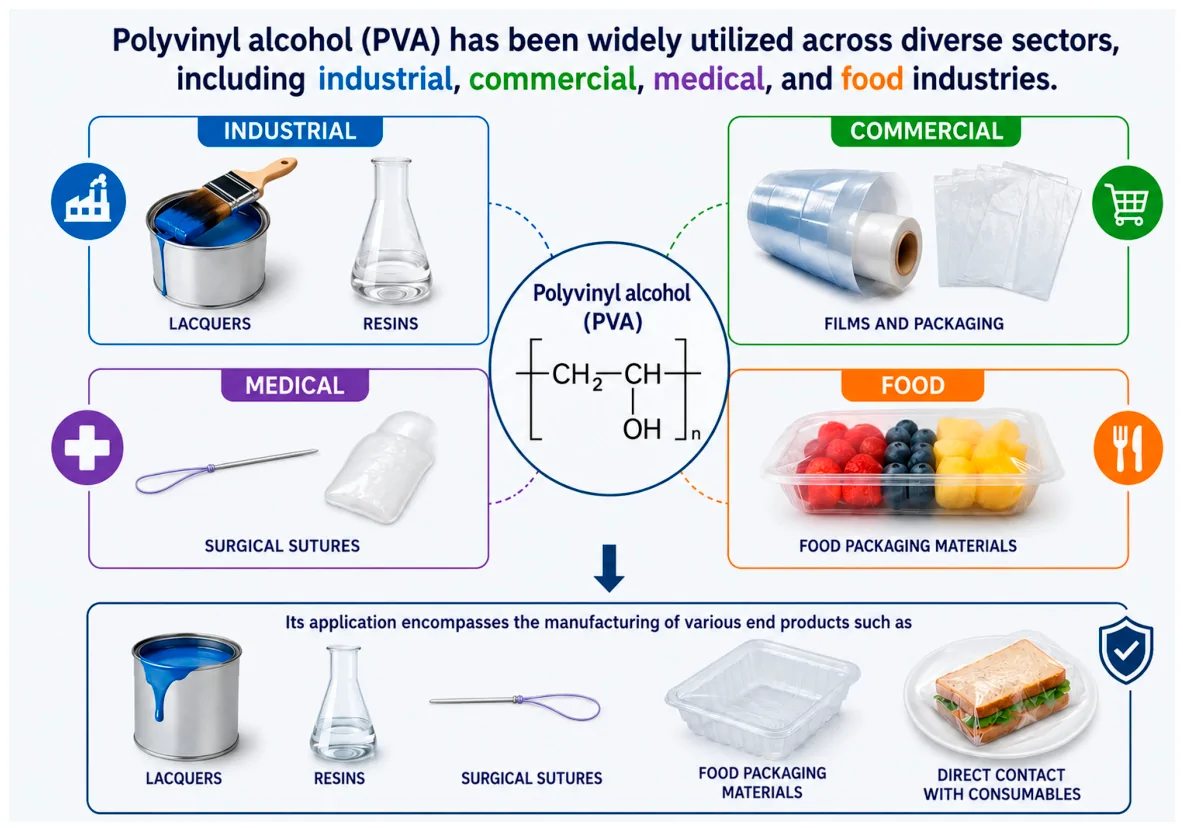
Infographic showing general applications of PVA in different sectors: industrial, commercial, medical, and food packaging.

Polyvinyl alcohol (PVA) has been widely utilized across diverse sectors, including industrial, commercial [29,62], medical [11,12], and food industries [31,63]. Its applications encompass the manufacturing of various end products such as lacquers, resins [64], surgical sutures [65], and food packaging materials [31,63], which frequently come into direct contact with consumables [11,15,17,21,66,67,68,69,70,71,72,73,74,75]. Therefore, PVA is the central point of extensive academic and industrial research due to its applications in crosslinked formulations and the incorporation of nanofillers, which enhance its functional properties and broaden its applicability [21,60,72]. In Figure 7, a schematic graphical illustration showing the different applications of PVA is presented.

PVA has been widely investigated for biomedical applications, including wound dressings, drug-delivery systems, and tissue engineering scaffolds. In controlled drug delivery, its biocompatibility, hydrogel-forming ability, and tunable matrix structure facilitate the incorporation and controlled release of therapeutic agents [76,77]. Its low protein adsorption, high water solubility, chemical resistance, and biocompatibility have also contributed to its use in a variety of medical devices, such as in orthopedics or soft contact lenses [69]. Depending on the intended application, the properties of PVA can be further tailored by blending it with other polymers or incorporating functional nanofillers. For example, Merkle et al. [78] investigated PVA/gelatin blends to promote endothelial cell adhesion and proliferation. More generally, the bioactivity, mechanical, and thermal properties of PVA-based materials can be modified by combining PVA with polymers such as poly(acrylic acid) (PAA), polyvinyl pyrrolidone (PVP), chitosan, and polyethylene oxide (PEO) [79,80,81].

To establish this framework, the following sections first examine the fundamental characteristics and biomedical performance of PVA hydrogels, followed by an overview of PVA nanofibers, with particular emphasis on the properties and functionalities that have been extensively demonstrated through electrospinning. Together, these two areas provide the scientific basis for evaluating the development of SBS-derived PVA nanofibers, and the opportunities and challenges associated with their biomedical applications.

### 3.1. PVA Hydrogels

**Figure 8:**
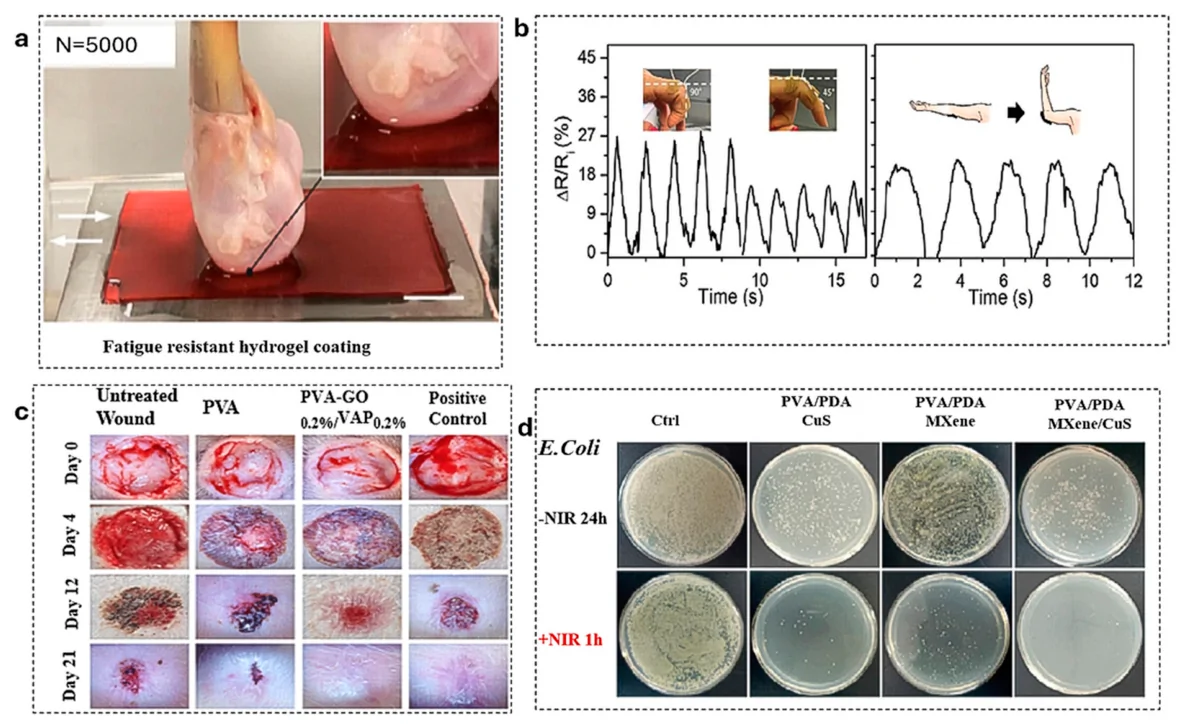
PVA hydrogel applications. (**a**) Artificial cartilage; reproduced with permission from [85]. (**b**) Wearable sensor; reproduced with permission from [87]. (**c**) Wound dressing; reproduced with permission from [91]. (**d**) Antimicrobial properties for wound dressings; reproduced with permission from [90].

PVA hydrogels have attracted significant attention in the healthcare sector due to their biocompatibility, tunable physicochemical properties, and adaptability for various biomedical applications [82]. These hydrogels can be explored for use in artificial cartilage [83,84,85], electronic skin [86,87], wound dressings [74,88,89,90,91], microneedling [92], and long-term sustained drug release for severe lesions [93] (Figure 8). The crosslinking of PVA hydrogels is a crucial factor that greatly affects their biological and mechanical properties. It enables researchers to accurately adjust the functionality of materials, resulting in a hydrogel characterized by a higher level of crosslinking, which provides a tighter network and decreases pore size while enhancing mechanical strength and elasticity. These characteristics are essential for controlled drug release and managing the adhesion of platelets in case of blood-contacting implants, ensuring implant stability and biocompatibility.

There are two primary methods of crosslinking utilized globally: physical and chemical. However, most approaches combine these two methods to strike a balance and achieve the desired properties [60]. The mechanical consistency required for the development of articular cartilage is much higher than that provided by a common PVA hydrogel. Li et al. [94] reported that natural cartilage has compressive and tensile strengths of 4 to 20 MPa and 0.8 to 25 MPa, respectively, whereas it has elasticity of 60% to 120% and a coefficient of friction (COF) of 0.001 to 0.04. It can transmit body weight up to 7–9 times and enables the human body to perform flexible activities for a longer time [62,94], whereas the pure PVA hydrogel can provide an ultimate tensile strength of 0.6 MPa and compressive strength of 24 MPa and can deform up to 20% due to a lower compression modulus (0.31–0.8 MPa) [84]. This obstacle is overcome by implementing different methods to develop a special PVA-based hydrogel for bionic joints. Increasing the crystallinity of the PVA hydrogel and introducing a multi-network hydrogel can have a positive impact on its mechanical properties. Multi-network hydrogels consist of interpenetrating polymer networks that collectively support the biological functionality and mechanical properties. This porous and three-dimensional framework favors ECM attachment and cell proliferation.

Ye et al. [71] prepared the composite hydrogel by physical crosslinking of polyethylene glycol (PEG)/PVA and achieved a mechanical strength of 21.2 MPa and a lower COF of 0.12 [71]. Solvent absorption in hydrogels can enhance the mechanical properties closer to those of natural cartilage [68]. Similarly, the addition of secondary networks such as polyacrylamide (PAAm), polyethylene glycol (PEG), chitosan, or alginate is another way to achieve strong interactions among components and enhance hydrogen bonding for improved mechanical properties. While chitosan (ChS) and other antibacterial biomaterials significantly improve bioactivity and mechanical strength [60,83,84], they may also increase brittleness [95]. Ye et al. [71] reported that graphene oxide-based tannic acid in a PVA hydrogel can enhance the tensile strength by 11–26 times compared to the pure PVA hydrogel. Furthermore, it was also observed that load-bearing capacity improved with solvent impregnation, resulting in a lower coefficient of friction (COF) of 0.12 under dry conditions. Solvent impregnation depends on the inherent structure of the highly porous and three-dimensional polymer hydrogel network, which can easily absorb and retain a large amount of solvent or liquid. This process achieves the required load-bearing capacity by ensuring the entire volume of material is lubricated. The inclusion of encapsulated carbon quantum dots (CDs) in a PVA hydrogel is another way to reduce friction [68]. These CDs act as molecular ‘ball bearings’, facilitating smooth sliding between the contact surfaces, or by forming a low-shear protective layer that minimizes polymer-to-surface contact. On the other hand, the addition of bioactive materials such as hydroxyapatite [96] and polysaccharides [97] to hydrogels can enhance cell adhesion and proliferation [98].

Hydrogels are widely used in wearable sensors to aid in disease diagnosis and the treatment process [86]. With their valuable characteristics of strain and temperature sensing, they are beneficial in the health sector [92,99]. Although the conductivity value of pure PVA-based hydrogel is lower, i.e., 4.24 × 10^−5^ S cm^−1^ [100], it can be enhanced by modifying it with different conductive nanoparticles, such as graphene [101], carbon nanotubes (CNTs) [102], polyaniline (PANi) [103], and charged ions of nanometallic particles [104]. Adding these conductive particles not only increases the electronic properties extraordinarily, but also, if employed well during the synthesis process (e.g., using the directional freezing method), enables directional responses to stimuli [60]. Hydrogels exhibit lower conductivity at temperatures below 0 °C, as water molecules crystallize, but their durability and flexibility decrease at higher temperatures. This is the major reason that binary solvents are mostly employed to prevent the loss of mechanical properties. Organic solvents such as ethylene glycol and dimethyl sulfoxide (DMSO), and glycerol [105] are commonly used as solvents to replace the water molecules in hydrogels. Dai et al. [106] prepared the hydrogel based on graphene oxide (GO)/PVA/polyacrylamide by using the binary solvent of ethylene glycol and water. It was observed that due to strong hydrogen bonding between water and the solvent, ice crystals were unable to form, which is why it could work smoothly at the lowest possible temperature (−50 to −80 °C), and it remained moist for up to 100 days. Wearable sensors need hydrogels with a functional power source and stretchability. To accommodate the power sources, frictional nano powder [107] and moisture generators [108] are commonly employed in PVA-based hydrogels [109]. Qin et al. [99] successfully developed a sensor to monitor real-time health by using sweat moisture to detect electrolytes and metabolites.

PVA hydrogels are extensively used in wound dressings due to their remarkable stretchability and a higher elastic modulus closely aligned with that of human natural skin [21,91]. The natural stretchability of skin is 60–70%, and the elastic modulus is 5–1000 kPa [110,111]. With these characteristics, antibacterial and anti-inflammatory properties can be incorporated using different active agents. PVA hydrogel, due to its flexibility and porosity (owing to the swollen network), is quite similar to the natural matrix of skin for wound coverage, with higher water content [107]. The inherent porosity of the PVA hydrogel network facilitates the formation of pathways through water-filled spaces and supports oxygen and nutrient diffusion, like blood vessels. These interconnected channels also serve as a passage for drug molecules. The porous structure and biodegradability of PVA hydrogel support accurate drug delivery and oxygen transport. The core focus of researchers is now to develop hydrogels with bioactive materials that can facilitate conditional drug release [13,112]. A prime example is the glucose-responsive hydrogel, which works conditionally and releases insulin only when blood glucose levels are high [113].

The crucial features to consider when dealing with chronic wounds with dressings are preventing bacterial infection and inflammation. Antimicrobial components such as copper sulfide (CuS), iron oxide (Fe_3_O_4_), etc., exhibit high efficacy in the presence of near-infrared light (NIR) irradiation [60]. Antibacterial strategies in PVA hydrogels generally fall into two categories: (i) direct incorporation of organic and natural polymers, (ii) loading of inorganic antimicrobial components. Inclusion of organic components such as chitosan [114], sodium alginate [115], and silk protein [116] in PVA wound dressings improves the healing process as well as the mechanical properties due to the antibacterial characteristics. Liu et al. [74] stated that due to the antimicrobial activities of chitosan-PVA hydrogel, it is being used for trauma dressings. Chitosan (ChS), with its unique characteristics, can cut down the nutrient supply of bacteria and interact with their membrane, and can destroy their DNA [75]. Organic antibacterial components can be incorporated into hydrogels; however, they often lose their antibacterial effectiveness due to inadequate crosslinking or bonding to support them. This is why ionic liquids are utilized to maintain antibacterial activity for an extended period [117]. Loading antimicrobial inorganic particles gives many advantages, as nanomaterials can perform antibacterial action through different mechanisms [118], such as photocatalysis, disruption of the cell membranes due to their smaller size, and the photothermal effect. Ultimately, they can damage bacterial enzymes and improve the healing process.

The microstructure of PVA hydrogel, with its superior features, such as higher water content, biocompatibility, and ability to encapsulate drugs, is essential for drug-delivery systems [119]. Huang et al. [120] also reported that a hydrogel consisting of gold nanoparticles (AuNPs) can respond to near-infrared resonance (NIR) stimulation and can remove up to 80% of bacteria. It was observed that bioactive peptide-functionalized AuNPs can promote diabetic healing through sequential drug release. Therefore, these unique hydrogels are also the focus of the health sector, as they can respond to the wound microenvironment and to substances that develop during the healing process. PVA hydrogel can be transformed into these smart hydrogels by using specific crosslinkers. Smart PVA hydrogels, capable of responding to external stimuli or wound microenvironments, have emerged as promising platforms for controlled therapy. Qiao et al. [121] reported that a physically crosslinked PVA-based smart hydrogel with a UV-responsive drug and modified silica nanoparticles can monitor minor bacterial infections and release the antibiotic drug via NIR.

Despite these advances, and although PVA hydrogels are highly promising for biomedical applications, their low mechanical strength and limited stability encourage the exploration of alternative approaches, such as PVA nanofibers, for long-term use [122,123].

### 3.2. PVA Nanofibers

PVA nanofibers are widely investigated for biomedical applications, including wound dressings [33,79], controlled drug-delivery systems [17,124], and tissue engineering scaffolds [14,15,125]. Their properties can be readily tailored through polymer blending, incorporation of functional components, and crosslinking, allowing fiber morphology, surface characteristics, mechanical performance, stability, and biological response to be adjusted according to the intended application. Aytimur and Uslu et al. [79], for example, investigated PVA/polyacrylic acid (PAA) nanofibers incorporating polyvinyl pyrrolidone (PVP-K30, where the K-value denotes a viscosity parameter correlated with molecular weight), polyvinyl pyrrolidone iodine complexes (PVP-I), and chitosan. This study revealed that the degradation temperature of the final nanofibers ranges from 400 °C to 450 °C, thereby confirming their thermal stability for potential applications in wound dressings that promote the formation of scar tissue. Incorporation of PVP and chitosan reduced the average fiber diameter from 458 to 270 nm, illustrating the influence of blend composition on fiber formation [79].

Reducing fiber diameter increases the specific surface area of the nanofibrous mat, which can influence drug-release behavior and interactions with the biological environment, which may significantly affect their application in drug delivery and wound healing [81]. In tissue engineering, control over fiber diameter is also important for reproducing structural features of the native extracellular matrix (ECM). More generally, blending PVA with chitosan or other polymers such as polyethylene oxide (PEO) or gelatin modifies solution properties, such as surface tension or viscosity [80], while physical or chemical crosslinking can be used to control the stability of the resulting nanofibrous structures [126].

In drug-delivery applications, nanofibrous systems generally rely on polymer swelling, diffusion, or degradation to release encapsulated therapeutic agents [127]. However, in the case of PVA, achieving sustained drug release is challenging due to its strong hydrophilicity, which often leads to rapid swelling and burst release. The controlled drug-release effect can be achieved by using nanofibrous composite membrane structures, such as those containing chitosan, alginate, glucan, etc. Zeng et al. [128] reported that the rapid dissolution of PVA nanofibers can facilitate efficient drug release, which is advantageous for applications requiring immediate therapeutic action. Conversely, when sustained release is required, stabilization of the highly hydrophilic PVA-based fibrous matrix becomes important. De Castro et al. [129] developed photo-crosslinked PVA/hyaluronic acid (HA) electrospun membranes as potential carriers for sustained drug delivery. Crosslinking improved the aqueous stability of the membranes while preserving their swelling capacity and cytocompatibility.

**Figure 9:**
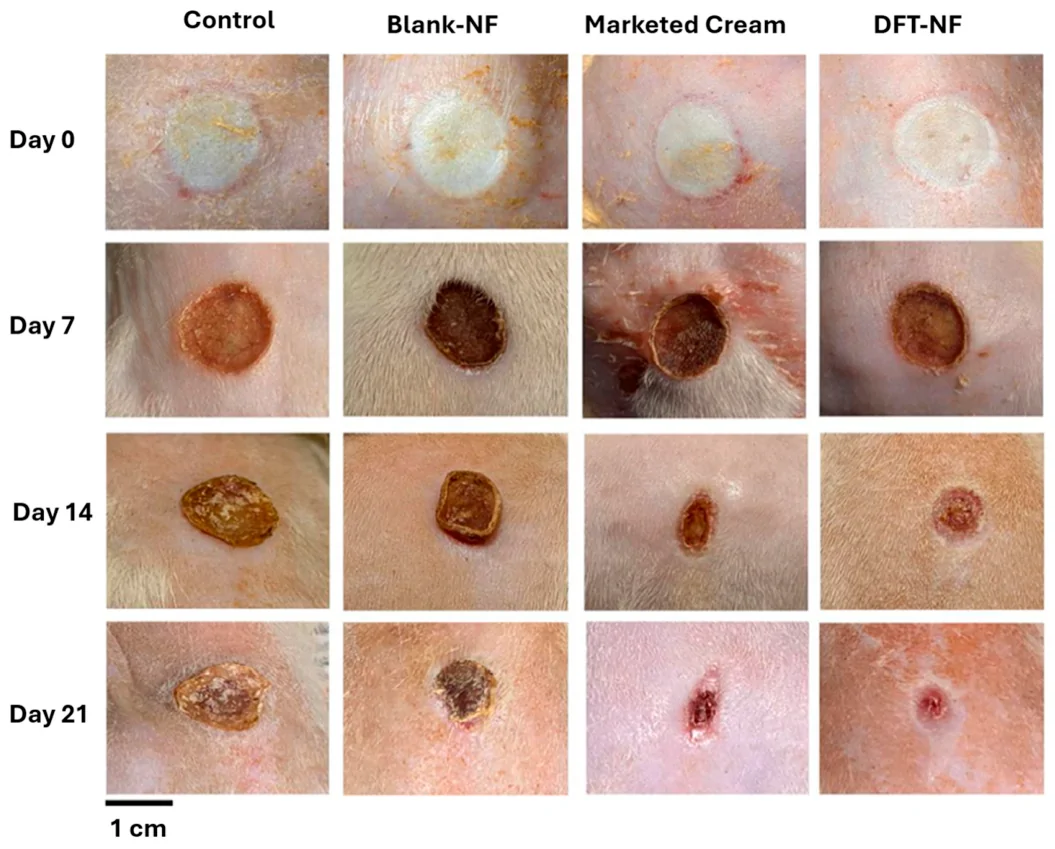
Burn wound closure analysis using the electrospun PVA/hyaluronic acid-based nanofibers (NF) encapsulating the Defactinib (DFT). Reproduced with permission from [130].

Similarly, PVA/HA electrospun nanofibers were fabricated by encapsulating Defactinib (DFT) for burn wound treatment [130]. The efficacy of these scaffolds was tested in vivo, and the progression of wound healing was monitored over a 21-day period (Figure 9). In the first 7 days, all experimental setups, including the marketed cream and drug-loaded scaffolds, showed similar behavior. However, by Day 14, differences became visible, with substantial wound healing due to tissue contraction. By Day 21, the DFT-NF presented full wound healing, showing near-complete wound closure, highlighting that the PVA/HA polymer matrix alone is not sufficient to treat thermal injury. Whereas in the case of DFT-NF, wound healing is related to the controlled drug release.

In addition, the preparation of polymers into nanofibers facilitates the development of complex and intricate microstructures using diverse polymer components, leading to an effective release mechanism. For example, coaxial electrospinning was employed to develop core–shell composite nanofibers with enhanced surface wetting and mechanical properties [131]. Alharbi et al. [132] investigated composite nanofibers consisting of PLA in the core and PVA in the shell, and reported the metabolic activities of human embryonic kidney cells and how they adhere to various nanofiber materials. The results of this study support the idea of using coaxial nanofibers in biomedical applications. The core/shell structure PLA/PVA nanofiber mat exhibited enhanced hydrophilic properties with a contact angle of 27 ± 15° as compared to 110 ± 2.5° for pristine PLA, thus favoring cell adhesion in biological environments. Moreover, the tensile strength and ductility of these coaxial nanofibers improved significantly, from 4.1 MPa and 40% to 14.5 MPa and 110%, respectively. These enhanced mechanical and surface properties make the coaxial nanofibers suitable for proliferation and cell attachment in biomedical applications.

Given the need for scaffolds with highly tailored properties, particularly mechanical strength and controlled volume for implantation, managing fluid dynamics is essential. Maintaining minimal volume changes can be achieved using pectin-chitosan scaffolds, which absorb liquid more quickly as compared to plain chitosan scaffolds, but with slightly slower cell growth. They exhibited tensile properties comparable to human natural skin [133]. Beyond structural and mechanical properties, the composition of PVA-based nanofibers can strongly influence cellular responses. Arsan et al. [134] studied the effect of composite electrospun nanofibers comprising PVA and polyhydroxybutyrate (PHB) on material properties and specific cell response. It was observed that the PVA content could accelerate the hydrolytic degradation of PHB. Interestingly, the cell line responded differently at different concentrations of the blend. Pure PHB supported proliferation and adhesion of the human keratinocyte cell line (HaCaT), but inclusion of 5 wt% PVA inhibited HaCaT growth. Conversely, a 50/50 ratio of PVA/PHB, significantly increased the HaCaT growth, simultaneously, due to increased hydrophilicity, fibroblast growth was completely inhibited due to reduced adsorption sites of cell attachment proteins. It was proposed that it is possible to have a bilayer fibrous structure with PVA/PHB (50/50) as a top layer to act as the epidermis to promote the growth of HaCaT and a layer with pure PHB or PVA/PHB (10/90) to attain the proper growth and adhesion of fibroblasts similar to the dermal layer.

Similarly, PVA/ChS blends can be used to create scaffolds with a large porous structure that support nervous tissue engineering and its repair. It was observed that the addition of ChS enhanced the growth and viability of nerve cells, thereby increasing the biocompatibility of PVA [135]. Ma et al. [136] reported the surface modification of PVA nanofibers with keratose nanoparticles and observed improved cyto-biocompatibility. This favors the idea that PVA nanofibers have potential for use in regenerative medicine. Zhou et al. [137] investigated the performance of carboxyethyl chitosan/PVA and PVA/silk fibroin nanofibers. These electrospun nanofibers demonstrated better in vitro biocompatibility during cytotoxicity testing with mouse fibroblasts.

In another study, a multilayer structure of PVA with polycaprolactone (PCL) was created to provide controlled drug release. Liu et al. [138] reported that a blend of PVA with gentamicin on the surface and its coaxial structure or PVA-desferoxamine (DFO)/PCL enhanced the controlled DFO drug release. Similarly, the combination of PVA’s hydrophilicity and PCL’s hydrophobicity makes it suitable for delivering water-soluble drugs and allows for the encapsulation and controlled release of a wider range of therapeutic agents, such as tetracycline hydrochloride and phenytoin sodium [139]. These facts support drug delivery for severe wounds and encourage the fast-healing process [139].

**Figure 10:**
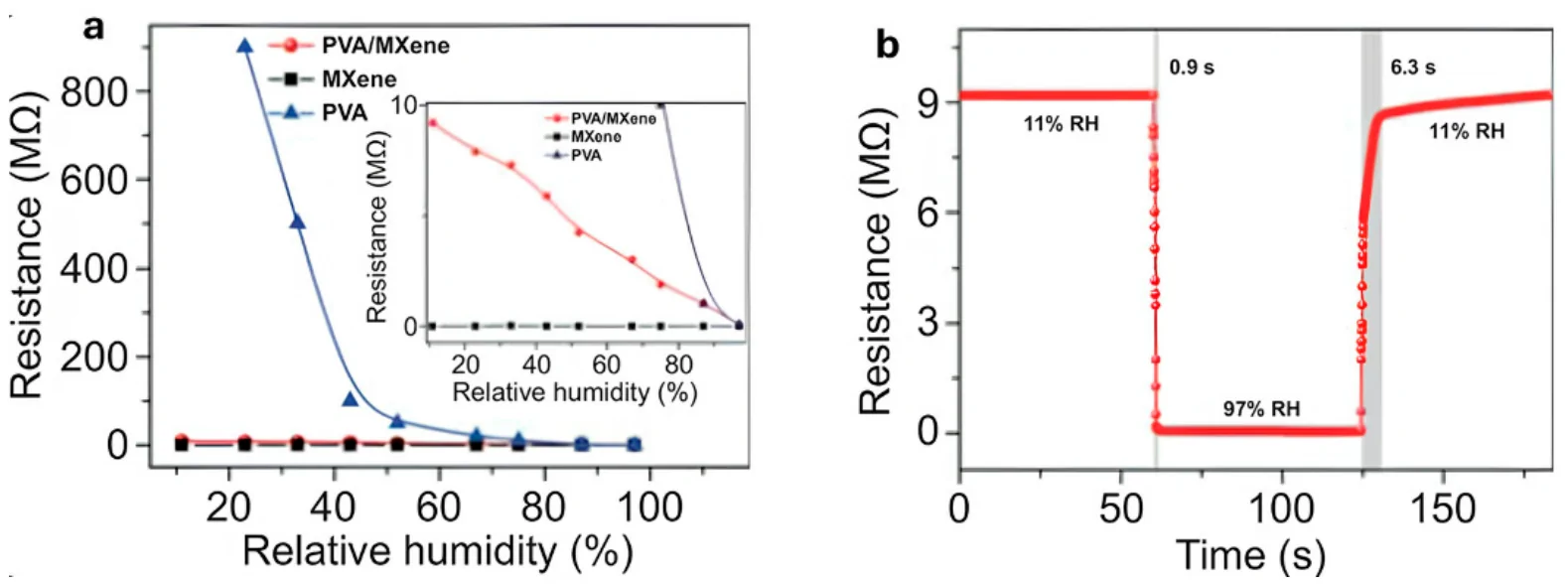
PVA/MXene sensors (**a**) Behavior of PVA/MXene sensors towards different humidities; (**b**) time-dependent response of sensors at 11% and 97% humidity (reproduced with permission from ref. [142]).

Shalumon et al. [33] developed the PVA/sodium alginate-based/zinc oxide (ZnO) nanofibers and stated that the incorporation of ZnO in PVA nanofibers not only increased fiber diameters but also inhibited the growth of bacterial strains. Nanofibrous materials are also useful for the fabrication of masks. Fathi-Azarbayjani et al. [140] observed in their study that nanofibers can be utilized to fabricate face masks. It is reported that the face mask of PVA/RMβ-CD (randomly methylated beta-cyclodextrin) can provide the required nutrients, including ascorbic acid, retinoic acid, gold, and collagen, to the skin, thereby enhancing its appearance. Similarly, biosensors are useful for the rapid analysis of diseases and for implementing preventive measures. In general, a biosensor consists of conductive nanofibers having a nanomesh of 300–700 nm with a water-soluble coating such as PVA. Firstly, gold is deposited on an electrospun PVA nanofiber mesh and laminated on the skin; then PVA is dissolved using water, and gold nanoparticles adhere to the skin and act as a sensor [141]. A flexible humidity sensor was prepared, comprising MXene and PVA nanofibers powered by a layer of molybdenum diselenide piezoelectric nanogenerator, and exhibited excellent electrical and sensing characteristics [142]. Figure 10 summarizes the humidity-sensing performance of PVA/MXene nanofiber-based devices, showing the dependence of resistance on relative humidity and the time-resolved response under alternating humidity levels. The hydrophilic nature of PVA and MXene helped to lower resistance (Figure 10).

Table 3 provides a broader selection of studies on electrospun PVA-based nanofibers, summarizing their composition, processing conditions, fiber characteristics, key outcomes, and biomedical applications. The studies are grouped according to their main application, including wound dressing, drug delivery, tissue engineering scaffolds, and wearable sensors. This overview illustrates the range of material formulations and processing strategies explored for electrospun PVA nanofibers and provides a reference framework for assessing the comparatively less developed field of SBS-fabricated PVA systems. 

Overall, the extensive research on electrospun PVA-based nanofibers demonstrates their versatility for biomedical applications and has established important relationships between composition, fiber architecture, and functional performance. However, the extent to which this knowledge can be transferred to PVA nanofibers produced by solution blow spinning remains comparatively unexplored, particularly given the distinct fiber-forming mechanisms and processing requirements of SBS.

## 4. Biomedical Applications of SBS-Fabricated PVA-Based Nanofibers

The use of solution blow spinning for the fabrication of PVA-based nanofibers has been investigated for a range of applications, with growing interest in biomedical systems [5,30,44,50,63,147,148,149,150,151,152,153,154,155]. In this context, the aqueous processability of PVA is particularly relevant, as it enables the fabrication of fibrous materials without relying on organic solvents, while also introducing specific challenges associated with water evaporation and fiber formation. Recent studies have explored different material formulations and processing strategies to obtain PVA-based fibrous structures for wound dressing, tissue engineering, antimicrobial applications, and drug delivery.

**Figure 11:**
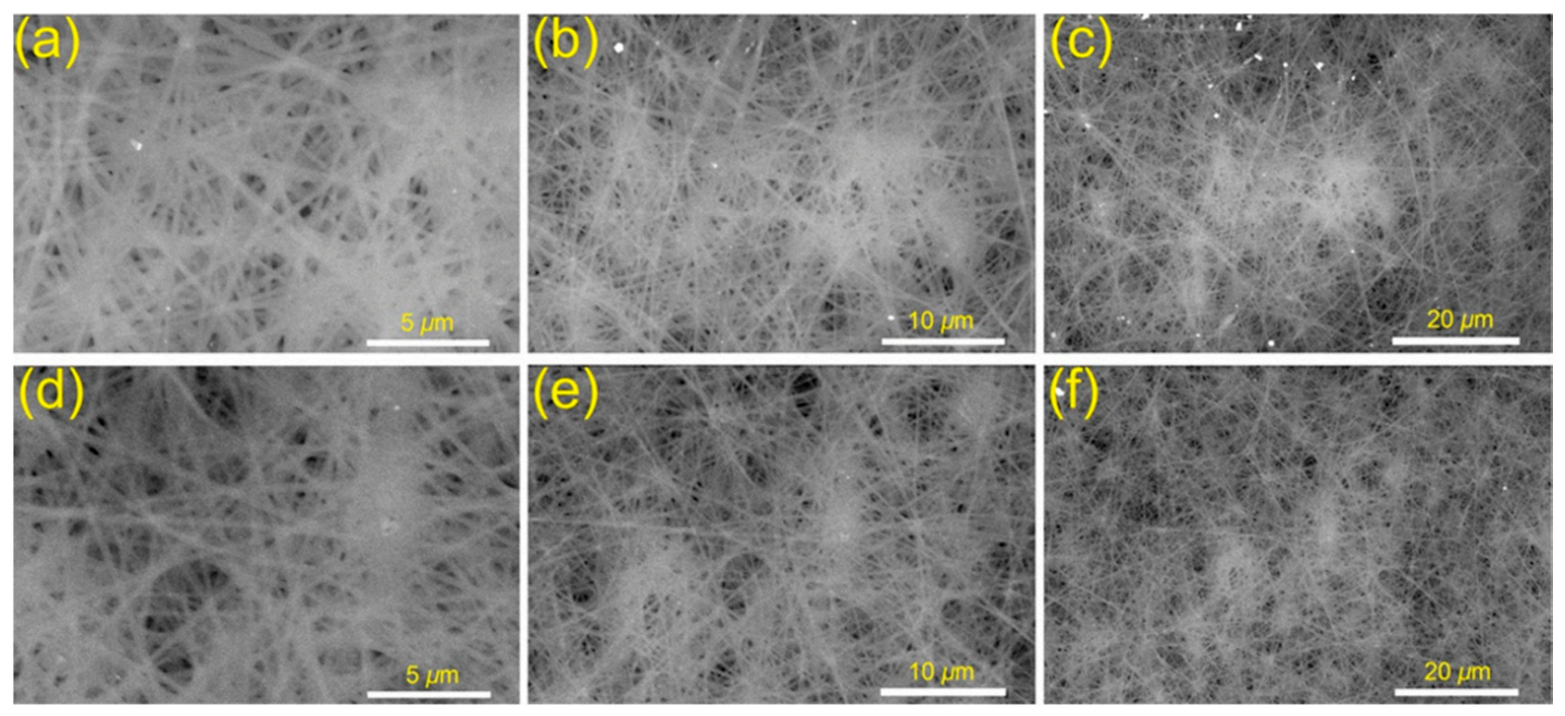
SEM images of solution-blown spun SP/PVA/ZnO nanofibers; (**a**–**c**) SP-0, and (**d**–**f**) SP-2; reproduced with permission from ref. [150].

Snari et al. [150] presented a novel approach to create biodegradable wound dressing nanofibers using sodium polylactate (SP), polyvinyl alcohol (PVA), and zinc oxide (ZnO) nanoparticles through solution blow spinning (SBS). This sustainable method and these materials for synthesizing the nanofibers aim to enhance wound healing. Sodium polylactate (SP), a biodegradable and biocompatible material, alongside PVA and ZnO, was used to produce the antibacterial nanofibers for wound dressings. Composite nanofibers of SP/PVA/ZnO were prepared by utilizing 0.5–4 wt% of ZnO, and the suspensions were pumped through the gauge needle at an air pressure of 0.5 bar and a feeding rate of 8 mL·h^−1^. The distance between the needle and collector was 25 cm. Samples were characterized using FTIR, NMR, SEM, and TEM to study the morphology and structure of the nanofibers. In Figure 11, the SEM micrographs for the ternary systems SP/PVA/ZnO obtained as a function of SP content (SP-0, SP-2) are shown. The images indicate a homogeneous, randomly oriented nonwoven fiber network (≈180–220 nm) for both SP-0 and SP-2, with no marked morphological change upon ZnO incorporation [150]. It was observed that nanofibers exhibited antibacterial activity against *Bacillus pumilus*, *Staphylococcus aureus*, *Klebsiella pneumoniae*, and *Escherichia coli,* even at the minimal concentration of ZnO, specifically, i.e., 0.5% and 2%. This study encourages and supports the idea of using SP/PVA/ZnO fibers for wound dressing to avert infections, with the advantage of maintaining biocompatibility [150].

One of the most significant recent advances in the biomedical application of SBS-processed PVA was reported by Cerqueira et al. [147], who developed PVA/chitosan (ChS) nanofibrous scaffolds through an environmentally friendly solution blow spinning process using water as the solvent. It was reported that the porous structure of the fibers is suitable for tissue engineering. Nanofibers were developed by using distilled water without any crosslinker, making them toxin-free. It is well known that the addition of chitosan not only enhances the viscosity and thermal properties but also helps to increase the antimicrobial properties and inhibits the growth of microorganisms. The average diameter of nanofibers decreased after the inclusion of CS, ranging from 566 ± 196 nm to 251 ± 47 nm. The resulting fibrous mats exhibited an interconnected porous network that closely resembles the architecture of the extracellular matrix, providing a favorable environment for cell adhesion, migration, and infiltration while facilitating the transport of nutrients and biological fluids. These results highlight the potential of aqueous SBS processing as a sustainable alternative for the fabrication of biomimetic scaffolds for tissue engineering.

Recently, Victor et al. [152] reported the use of the air-heated SBS technique to fabricate the 3D nanofibrous structure of PVA/ChS, and it was observed that the thermal stability of nanofibers decreased due to weakened intermolecular bonding, whereas the porosity was increased up to 80% in the case of PVA/ChS. Excessive ChS dosage resulted in a rough surface and ultimately in bead formation. But the biocompatibility and highly porous structure of fibers allow them to be used in wound dressing and tissue engineering.

**Figure 12:**
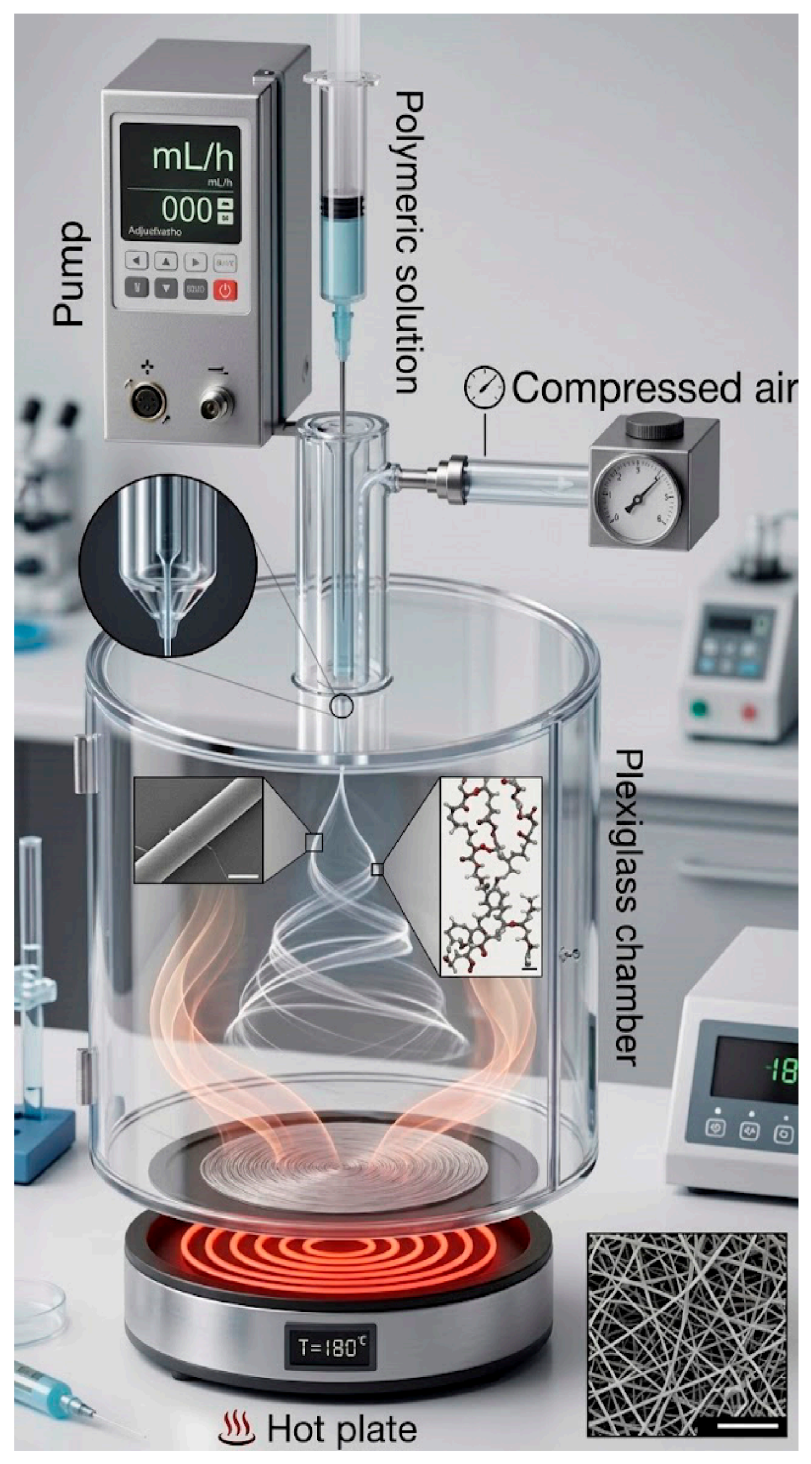
Schematic of heat-assisted setup for solution blow spinning, adapted from ref. [148].

Scaffaro et al. [148] also used the heat-assisted SBS (Figure 12) technique to develop crosslinked PVA nanofibers incorporating graphene nanoplatelets (GNP) and chlorhexidine (CHX). It was observed that GNPs improved the mechanical properties and crosslinking of nanofibers, as well as their antimicrobial activity against Gram-positive and Gram-negative bacteria. Processing conditions for the fabrication of nanofibers were as follows: a flow rate of 45 mL·h^−1^, air pressure of 0.5 MPa, a needle-to-collector distance of 20 cm, a spinning time of 15 min, and a collector plate temperature of 180 °C to introduce thermal crosslinking. To understand the effect of thermal crosslinking, samples were analyzed through FTIR, SEM, and DSC. It was observed that the PVA/CHX-GNP provided smoother fibers with higher stability in water after immersion for 45 h [148]. This study points out the use of thermal crosslinking in the presence of GNPs as an effective method to prepare optimized materials for controlled drug release applications.

Although biomedical applications constitute the main focus of this review, the broader SBS-PVA literature provides important information on the processability and functional versatility of PVA. Table 4 summarizes the current state of the art of poly(vinyl alcohol) (PVA) processed by solution blow spinning (SBS). The available literature demonstrates that, despite the versatility of PVA, research on SBS processing remains limited, with only a small number of studies reported to date. Initial investigations focused on establishing the feasibility of producing PVA nanofibers from aqueous solutions, whereas more recent studies have expanded toward biomedical applications, particularly wound dressings and tissue engineering, taking advantage of the polymer’s biocompatibility, hydrophilicity, and ease of functionalization. Beyond the biomedical field, PVA has also been explored in environmental remediation, advanced engineering materials, and sustainable food packaging, where it serves as either a matrix polymer, blending component, or sacrificial processing aid. Overall, these studies demonstrate the versatility of PVA in SBS while highlighting that biomedical applications remain an emerging research area with considerable opportunities for further development. Comparison of the PVA-SBS studies summarized in Table 4 with the electrospun PVA systems presented in Table 3 indicates that electrospinning-derived processing conditions cannot be directly extrapolated to SBS because the two techniques rely on distinct fiber-forming mechanisms. 

All these studies indicate a progression in the use of PVA in SBS. Early investigations focused primarily on establishing the feasibility of producing PVA fibers from aqueous solutions, for which the relatively low volatility of water represents an important constraint on solvent removal during fiber formation. This has led to the use of thermally assisted approaches, including heated air and heated collection systems, to enhance water evaporation and facilitate fiber formation. Subsequent studies have investigated more complex formulations combining PVA with other polymers, nanoparticles, antimicrobial agents, and bioactive compounds to modify fiber morphology, stability, mechanical properties, and functionality. These developments have extended the role of PVA in SBS from the investigation of aqueous fiber formation toward the fabrication of functional fibrous materials for different applications.

For biomedical applications, the hydrophilicity and water solubility of PVA have important implications for both processing and subsequent material performance. While aqueous processing avoids the use of organic solvents, stabilization of the resulting fibers may be required to preserve their structure upon exposure to aqueous environments. Polymer blending, incorporation of functional components, and thermal crosslinking have been investigated as strategies to control fiber properties and introduce antimicrobial or drug-delivery functionality. Nevertheless, the available SBS literature is still largely focused on processing, morphology, physicochemical characterization, and antimicrobial performance, whereas biological validation remains comparatively scarce. Further investigation of cell–material interactions, degradation behavior, controlled release, and in vivo performance would therefore help establish the relationship between SBS processing, PVA nanofiber structure, and biomedical functionality.

Within the biomedical field, the available studies demonstrate the potential of SBS-fabricated PVA-based fibers for wound dressing, tissue engineering, antimicrobial materials, and drug-delivery applications. However, the evidence reported to date is predominantly based on fiber morphology, physicochemical properties, water stability, and antimicrobial performance, with biological validation addressed in a smaller number of studies. Thus, while the existing literature establishes a basis for the development of functional PVA-based materials by SBS, further progress requires a better understanding and control of the processing and material-related factors that determine fiber formation, stability, reproducibility, and subsequent biomedical performance. These challenges are discussed in the following section.

## 5. Challenges and Limitations

Poly(vinyl alcohol) (PVA) nanofibers exhibit many attractive properties for biomedical applications; however, they also present limitations related to crosslinking efficiency, solubility, stability, and swelling behavior [148]. In particular, the intrinsic mechanical strength of PVA nanofibers is relatively low, often necessitating additional reinforcement or chemical modification to meet application-specific requirements. In the context of solution blow spinning (SBS), a key challenge lies in achieving effective solvent evaporation during fiber formation. Complete solvent removal is essential to ensure that only the polymer reaches the collector, where pre-shaped fibers can subsequently be crosslinked without the use of toxic chemicals, which is critical for biomedical applications. To address this issue, researchers have explored strategies to enhance solvent evaporation prior to or during fabrication, such as applying heat or light sources to raise the temperature within the spinning chamber. If these challenges can be effectively addressed, SBS may enable the production of a wide range of biodegradable nanofibers through a sustainable and environmentally friendly process.

Conventional electrospinning is generally well suited to polymers dissolved in volatile organic solvents, such as chloroform or acetone, which evaporate rapidly during jet flight. In contrast, poly(vinyl alcohol) (PVA) is a hydrophilic polymer that is typically processed from aqueous solutions. However, water possesses a substantially higher boiling point and lower vapor pressure than most organic solvents, resulting in a considerably slower evaporation rate. In electrospinning, this limitation is partially mitigated by the relatively low solution flow rates (typically 0.1–0.8 mL h^−1^) and short working distances (7–25 cm), which provide sufficient residence time for progressive solvent evaporation under ambient conditions before the fibers reach the collector [17,28,80,143,144,145,146].

This limitation becomes considerably more critical in solution blow spinning (SBS). Depending on the processing conditions, solution feeding rates typically increase to 1.2–45 mL h^−1^ [63,147,148,149,151,152,156,157,158,159,160,161], while the polymer jet is accelerated by a high-velocity gas stream generated at pressures of up to 0.68 MPa [151] over working distances as long as 80 cm [147]. Although the working distance may be greater than that used in electrospinning, the extremely high gas velocity reduces the jet residence time to only a few milliseconds. Such a short flight time is generally insufficient to achieve complete water evaporation under ambient conditions, particularly for concentrated PVA solutions, thereby preventing adequate fiber solidification before deposition. Consequently, the partially solidified fibers tend to coalesce upon reaching the collector, producing dense, non-porous films instead of well-defined fibrous mats.

Several strategies have been proposed to overcome this limitation by accelerating solvent evaporation. These include preheating the air stream, maintaining the spinning chamber at temperatures between 45 and 70 °C [147,149,152,156,157], using heated air streams at 40 ± 2 °C [63], and implementing heat-assisted SBS systems combining chamber temperatures of 60 °C with collector temperatures as high as 180 °C [148]. While these approaches have significantly improved fiber formation from aqueous PVA solutions, their effectiveness remains largely empirical. To date, no quantitative framework has been established to describe the coupled influence of solvent evaporation kinetics, ambient humidity, gas velocity, solution concentration, and thermal conditions on fiber formation during SBS. The development of such predictive models would not only facilitate process optimization but also improve the reproducibility and scalability of SBS for aqueous polymer systems.

Although PVA is highly attractive because of its excellent water solubility and biocompatibility, these same characteristics limit its direct application in environments where prolonged exposure to aqueous media is required. Consequently, applications such as wound dressings, water filtration membranes, and agricultural matrices generally require the polymer network to be stabilized through crosslinking. Current crosslinking strategies largely rely on post-processing treatments involving chemical agents, such as glutaraldehyde, or thermal curing. While these approaches effectively improve water resistance and structural stability, they partially compromise the environmental advantages associated with water-based SBS processing and may introduce concerns regarding cytotoxicity, residual crosslinking agents, or increased energy consumption. Consequently, the development of environmentally benign crosslinking strategies based on naturally derived agents, such as citric acid [126,163], represents one of the most promising research directions. In particular, crosslinking systems capable of being activated in situ during fiber formation could eliminate the need for additional post-processing treatments while preserving the sustainability of aqueous SBS. However, achieving sufficiently rapid crosslinking within the millisecond residence time of the SBS jet remains a major scientific challenge that has yet to be addressed systematically.

The rheological behavior of aqueous PVA solutions represents another major challenge for SBS processing. These solutions exhibit pronounced viscoelasticity and elastic recovery under the high shear rates experienced during extrusion, frequently leading to die swell phenomena that considerably complicate jet dynamics. Despite these complexities, most published studies employ custom-built concentric nozzles or modified commercial airbrushes, with relatively limited information regarding the influence of nozzle geometry on fiber formation. Since the polymer solution is entirely surrounded by a high-velocity annular air stream, even small variations in internal needle diameter, wall thickness, bevel angle, or protrusion length may significantly modify the local aerodynamic field and consequently affect jet stretching and fiber morphology. Nevertheless, no standardized nozzle geometries or design guidelines have yet been established for SBS processing of viscoelastic aqueous polymers. This limitation is further illustrated by the broad range of operating conditions reported in the literature, including air pressures between 0.001 MPa [158] and 0.68 MPa [151], working distances from 15 cm [161] to 80 cm [147], and solution flow rates ranging from 0.6 mL h^−1^ [161] to 45 mL h^−1^ [148]. Consequently, direct comparison among studies remains difficult, and the reproducibility of fiber morphology across different laboratories is still a critical challenge [44].

Beyond laboratory-scale optimization, industrial implementation of SBS introduces additional aerodynamic challenges. One of the principal advantages frequently attributed to SBS over electrospinning is its considerably higher production rate and its potential for straightforward scale-up. However, transitioning from a single-nozzle laboratory configuration to industrial multi-nozzle systems fundamentally changes the fluid dynamics of the process. Closely spaced high-velocity air jets generate complex turbulent flow fields and vortex interactions that may cause neighboring polymer jets to interfere with one another. For highly viscoelastic aqueous systems such as PVA, these interactions may promote fiber entanglement, jet instability, non-uniform mat deposition, and local variations in fiber diameter. Although computational fluid dynamics and experimental aerodynamic studies have begun to address these issues, systematic investigations of multi-nozzle SBS configurations remain scarce. Consequently, the lack of predictive design criteria for large-scale nozzle arrays continues to represent one of the principal barriers limiting the industrial translation of SBS technology [44].

**Figure 13:**
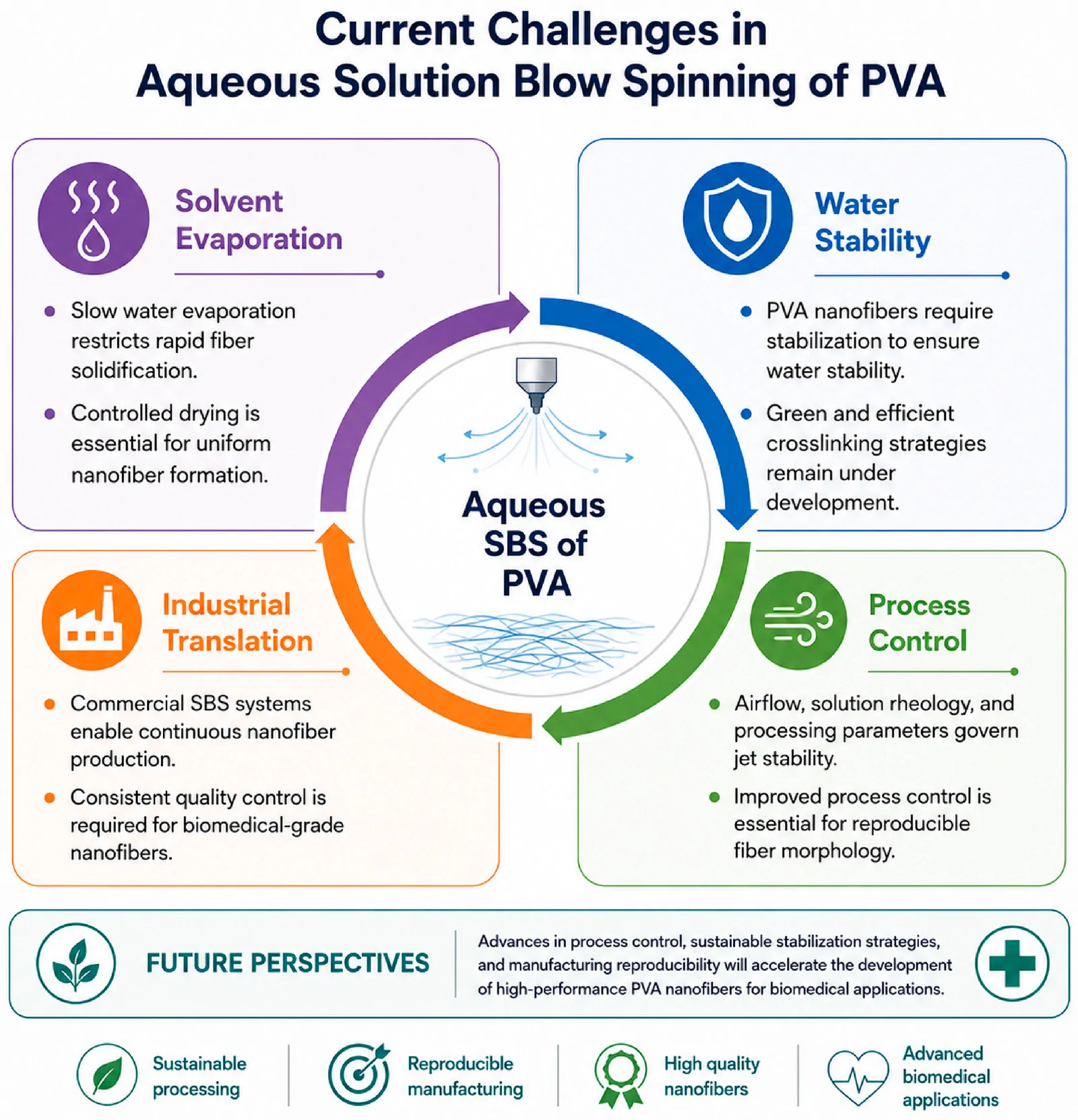
Current challenges and future perspectives for aqueous solution blow spinning (SBS) of poly(vinyl alcohol) (PVA).

Overall, the PVA-SBS studies summarized in Table 4 employed polymer concentrations of approximately 8–20%. This interval should not be interpreted as a universal processing window, since the concentration basis varies among studies and spinnability and fiber morphology depend jointly on molecular weight, degree of hydrolysis, solvent composition, and solution rheology. Meaningful cross-laboratory comparison therefore requires systematic reporting of these material and processing variables, together with nozzle geometry, gas pressure and flow rate, working distance, temperature, relative humidity, and collector configuration. The principal challenges identified and the corresponding future research directions for aqueous PVA-SBS are summarized in Figure 13.

## 6. Conclusions and Future Perspectives

This review examines the development of PVA-based nanofibers fabricated by solution blow spinning, placing the emerging SBS literature within the broader knowledge established for PVA hydrogels and electrospun nanofibers. The available studies demonstrate that SBS can produce PVA-based fibrous materials for biomedical applications, including wound dressings, tissue-engineering scaffolds, antimicrobial materials, and drug-delivery systems. Nevertheless, the biological evidence remains predominantly limited to in vitro evaluations; consequently, these applications remain exploratory and cannot yet be considered preclinically validated. At the same time, PVA represents a particularly relevant model for investigating the processing of aqueous polymer solutions by SBS, where the low volatility of water, the viscoelastic behavior of concentrated polymer solutions, and the short residence time of the accelerated jet impose specific requirements on fiber formation and solidification.

Recent developments in thermally assisted SBS, polymer blending, functionalization, and crosslinking have expanded the range and functionality of PVA-based fibrous systems. Nevertheless, several aspects require further investigation. In particular, establishing quantitative relationships between solution properties, solvent evaporation, processing conditions, and fiber morphology would provide a stronger basis for process optimization and reproducibility. Further development of biocompatible stabilization strategies is also required for applications involving prolonged exposure to aqueous environments, together with systematic investigation to develop standardized protocols to accelerate regulatory approval and commercial translation. From a biomedical perspective, more extensive biological validation, including cell–material interactions, degradation behavior, controlled-release studies, and *in vivo* evaluation, will be necessary to establish the functional performance of SBS-fabricated PVA systems beyond their physicochemical characterization.

In this context, the significance of PVA extends beyond its individual biomedical applications. Its combination of biomedical relevance, aqueous processability, and demanding fiber-formation behavior makes PVA a useful reference system for advancing the understanding of aqueous solution blow spinning. Progress in controlling these processing–structure–property relationships may therefore contribute not only to the development of PVA-based biomedical nanofibers, but also to the broader implementation of SBS for water-processable polymer systems. Future studies should combine standardized in vitro cytocompatibility and degradation assays with appropriately controlled, application-specific in vivo models, reporting quantitative endpoints such as cell adhesion and viability, degradation kinetics, inflammatory response, tissue integration, and functional recovery to support clinical translation.

## Figures and Tables

**Table 3:** Representative biomedical applications and processing parameters of electrospun PVA-based nanofibers.

Material (Composition)	Electrospinning Conditions	Diameter (nm)	Key Characterization /Outcomes	Application
PVA/ChS Thiourea/Ag -PHMBG (Polyhexamethylene biguanide) [143] 8 wt%/3 wt%/4 wt%	V = 16 kV; WD = 16 cm; FR = 0.5 mL h^−1^; Heat treatment, 130 °C—20 min	161 ± 34	Young’s modulus: 0.40 ± 0.02 MPa; Tensile Strength: 3.24 ± 0.31 MPa; WVTR 4.4 ± 0.47 kg·m^−2^	To Heal 2nd-Degree Burn Wounds
PVA/ChS/CuO [144] (10 wt%/3 wt%/10 wt%)	V = 15 kV; WD = 15 cm FR = 0.8 mL h^−1^	86 ± 20	Inhibition zones *E. coli* 22 mm *S. aureus* 19 mm	Wound Dressing
PVA/ChS/TCH (Tetracycline hydrochloride) [145] 10 wt%/3 wt%/5 wt%	V = 15 kV; WD = 15 cm FR = 0.1–0.35 mL h^−1^; T = 37 °C; RH = 40%	119 ± 33	Inhibition zones *E. coli* 8.8 ± 0.4 mm, *S. epidermidis* 15.6 ± 0.3 mm, *S. aureus* 19.6 ± 0.2 mm	Wound Dressing
PVA/ChS/ZnO [146] 8 wt%/3 wt%/0.014 wt%	V = 15 kV; WD = 7 cm FR = 0.5 mL h^−1^	279 ± 7	Inhibition zones *E. coli*: 20.2 ± 1.0 mm, *P. aeruginosa* 21.8 ± 1.5 mm, *B. subtilis* 15.5 ± 0.8 mm, *S. aureus* 21.5 ± 0.5 mm	Wound Dressing
PVA/ChS/GNPs (Graphene nanoplates)-loaded with Erythromycin [28] 8 wt%/3 wt%/10 wt%	V = 20 kV; WD = 11 cm FR = 0.1 mL h^−1^ T = 37 °C	216 ± 14	Inhibition zones *Staphylococcus aureus* 15.5 mm; *Pseudomonas aeruginosa* 13.5 mm; Drug release of 75.13% after 72 h	Drug Delivery
PVA/ChS/Ampicillin Sodium [17] 10 wt%/3 wt%/10 wt%	V = 15 kV; WD = 15 cm FR = 0.5 mL h^−1^ T = 50 °C; RH = 40%	287 ± 10	Young’s modulus: 198.1 MPa Tensile strength: 4.6 MPa	Transdermal Drug Release
PVA/MXene [80] 10% (*w*/*v*)/0.2% (*w*/*v*)	V = 20 kV; WD = 25 cm; FR = 0.3 mL h^−1^; T = 37 °C	587 ± 191	Young’s modulus: 136.87 ± 19.63 MPa Tensile strength: 7.35 ± 0.78 MPa	Tissue Scaffolds, Wearable Sensors

Abbreviations: V = voltage; WD = working distance; FR = feeding rate; T = temperature; RH = relative humidity; WVTR = water vapor transmission rate.

**Table 4:** Current state of the art of poly(vinyl alcohol) processed by solution blow spinning (SBS).

Application	Function of PVA/ Processing Conditions	Main Contribution
**A. Foundational processing**		
Aqueous SBS of PVA micro-/nanofibres [156]	Matrix polymer; AP = 0.55 MPa; WD = 30 cm; FR = 7.2 mL h^−1^ (0.12 mL·min^−1^); T_chamber_ = 45 °C	***First demonstration of aqueous SBS of PVA***. Fiber formation from water was enabled using heated air, while fiber diameter was governed by polymer concentration and molecular weight.
PVA/zein composite nanofibers [30]	Matrix polymer; AP = 0.48 MPa; WD = 40 cm; FR = 5.0 mL h^−1^ (0.083 mL·min^−1^); T_needle_ = 45 °C	**Production and characterization of PVA/zein nanofibers by SBS;** influence of zein concentration on fiber morphology, thermal stability, and mechanical properties explored. Food and biomedical applications.
**B. Biomedical applications**		
Wound dressing nanofibrous films [150]	Matrix polymer; (SP/PVA/ZnO); AP = 0.5 MPa; WD = 25 cm; FR = 7.8 mL h^−1^ (0.13 mL·min^−1^)	SBS was used to produce ***SP/PVA/ZnO nanocomposite*** wound dressings via a green, sustainable approach. ZnO nanoparticles provided antimicrobial functionality while PVA contributed flexibility and biocompatibility to the fibrous film.
Wound dressing/drug delivery [151]	Matrix polymer; (PVA/zein); AP = 0.68 MPa; WD = 60 cm; FR = 6.0 mL h^−1^ (0.1 mL·min^−1^)	***Biocompatible fibrous mats*** with sustained α-tocopherol release, improved mechanical performance, and cell viability above 90%; functional wound dressings.
Tissue engineering [147]	Matrix polymer; (PVA/chitosan); AP = 0.55 MPa; WD = 80 cm; FR = 6.0 mL h^−1^ (0.1 mL·min^−1^); T_chamber_ = 45 °C	***Green aqueous fabrication of porous PVA/chitosan nanofibres without crosslinkers***. Chitosan improved thermal stability while reducing water uptake and swelling, producing suitable scaffold-like structures.
Tissue engineering [152]	Matrix polymer; (CS/PVA and CS/PLA blends to produce 3D structures); AP = 0.48 MPa; WD = 40 cm; FR = 6.0 mL h^−1^ (0.1 mL·min^−1^); T_chamber_ = 70 °C	***3D nanofibrous structures*** from high-content chitosan/PVA and chitosan/PLA blends using air-heated solution blow spinning (A-HSBS). Demonstrated scalability of heated-air SBS for chitosan-rich biomedical fiber fabrication.
Bone tissue engineering [157]	Sacrificial spinning agent; AP = 0.41 MPa; WD = 50 cm; FR = 6.0 mL h^−1^ (0.1 mL·min^−1^); T_chamber_ = 45 °C	***Bioactive glass scaffolds were fabricated using PVA as a processing aid***. Rapid hydroxyapatite formation in simulated body fluid and cytocompatibility with MC3T3-E1 cells demonstrated the potential for bone regeneration.
Antimicrobial membranes [148]	Matrix polymer; (Heat-Assisted Process); AP = 0.5 MPa; WD = 20 cm; FR = 45 mL h^−1^ (0.7 mL min^−1^) T_collector_ 180 °C; T_chamber_ 60 °C	***Heat-assisted SBS:*** enabled one-step production of crosslinked PVA fibers incorporating chlorhexidine and graphene nanoplatelets. Water stability, controlled drug release, and broad-spectrum antimicrobial activity were achieved.
**C. Environmental & engineering applications**		
Magnetic composite fibers [149]	Sacrificial spinning agent AP = 0.41 MPa; WD = 60 cm; FR = 3.0 mL h^−1^ (0.041 mL·min^−1^); T_chamber_ = 60 °C	Ni/NiO-carbon magnetic fibers obtained after calcination of SBS precursors; PVA served exclusively as the spinning template.
Superconducting nanowires [158]	Sol–gel Carrier; AP = 0.001 MPa; WD = 40 cm; FR = 3.0 mL h^−1^ (0.05 mL·min^−1^) Halogen light above working distance	PVA enabled fabrication of YBaCuO nanowire mats through SBS before being removed during thermal processing.
Radionuclide capture [159]	Matrix polymer; AP = 0.1 MPa; WD = 40 cm; FR = 1.2 mL h^−1^ (0.02 mL·min^−1^)	Functional chitosan/PVA composite nanofibres with high uranium adsorption capacity, good recyclability, and antibacterial activity for wastewater remediation.
Solar desalination & water harvesting [160]	Hydrogel matrix; AP = 0.02–0.03 MPa; FR = (0.05–0.06 mL·min^−1^)	Hierarchical hydrogel sub-microfibres with excellent solar evaporation and atmospheric water harvesting performance.
**D. Food science & packaging**		
Active postharvest coatings [63]	Matrix polymer; AP = 0.48 MPa; WD = 40 cm; FR = (0.06 mL·min^−1^) Air temperature 40 ± 2 °C	***PVA nanofibres incorporating plant extracts*** with antioxidant activity for extending strawberry shelf life.
Food packaging [161]	Blending polymer; AP = 0.2–0.4 MPa; WD = 15–40 cm; FR = 0.6–6 mL·h^−1^ (0.01–0.1 mL·min^−1^)	Review describing ***PVA as a blending component*** to improve the spinnability of animal-derived proteins for sustainable food packaging.
**E. Other**		
Enzyme-functionalized nonwovens. Doctoral dissertation “Enzyme Functionalized Solution Blown Nonwovens” [162]		

Abbreviations: AP = air pressure; AF = air flow; WD = working distance; FR = feeding rate; T = temperature; RH = relative humidity.

## Data Availability

No new data were created or analyzed in this study. Data sharing is not applicable.

## Abbreviations

The following abbreviations are used in this manuscript:

**Abbreviation**	**Definition**
ChS	Chitosan
CHX	Chlorhexidine
COF	Coefficient of friction
CQD	Carbon quantum dot
CS	Centrifugal spinning
CuO	Copper oxide
DFO	Desferoxamine
DMSO	Dimethyl sulfoxide
DSC	Differential scanning calorimetry
ECM	Extracellular matrix
EMI	Electromagnetic interference
ES	Electrospinning
FR	Feeding rate
FTIR	Fourier transform infrared spectroscopy
GNP	Graphene nanoplatelets
GO	Graphene oxide
HA	Hyaluronic acid
MS	Melt spinning
NIR	Near-infrared light
NMR	Nuclear magnetic resonance
PAA	Poly(acrylic acid)
PAAm	Polyacrylamide
PCL	Polycaprolactone
PEG	Poly(ethylene glycol)
PEO	Poly(ethylene oxide)
PHB	Polyhydroxybutyrate
PHMBG	Polyhexamethylene biguanide
PLA	Poly(lactic acid)
PVA	Poly(vinyl alcohol)
PVP	Polyvinyl pyrrolidone
RH	Relative humidity
RMβ-CD	Randomly methylated β-cyclodextrin
SBS	Solution blow spinning
SEM	Scanning electron microscopy
SP	Sodium polylactate
TCH	Tetracycline hydrochloride
TEM	Transmission electron microscopy
TGA	Thermogravimetric analysis
UV	Ultraviolet
WD	Working distance
ZnO	Zinc oxide
WVTR	Water vapor transmission rate

## References

[1] Ashraf R., Sofi H.S., Malik A., Beigh M.A., Hamid R., Sheikh F.A. (2019). Recent Trends in the Fabrication of Starch Nanofibers: Electrospinning and Non-Electrospinning Routes and Their Applications in Biotechnology. Appl. Biochem. Biotechnol..

[2] Huang Z.-M., Zhang Y.Z., Kotaki M., Ramakrishna S. (2003). A Review on Polymer Nanofibers by Electrospinning and Their Applications in Nanocomposites. Compos. Sci. Technol..

[3] Kim S., Min S., Choi Y.S., Jo S.-H., Jung J.H., Han K., Kim J., An S., Ji Y.W., Kim Y.-G. (2022). Tissue Extracellular Matrix Hydrogels as Alternatives to Matrigel for Culturing Gastrointestinal Organoids. Nat. Commun..

[4] Kakoria A., Sinha-Ray S. (2018). A Review on Biopolymer-Based Fibers via Electrospinning and Solution Blowing and Their Applications. Fibers.

[5] Carriles J., Nguewa P., González-Gaitano G. (2023). Advances in Biomedical Applications of Solution Blow Spinning. Int. J. Mol. Sci..

[6] Croitoru A.M., Ficai D., Ficai A., Mihailescu N., Andronescu E., Turculet S.C. (2020). Nanostructured Fibers Containing Natural or Synthetic Bioactive Compounds in Wound Dressing Applications. Materials.

[7] Liu M., Duan X.P., Li Y.M., Yang D.P., Long Y.Z. (2017). Electrospun Nanofibers for Wound Healing. Mater. Sci. Eng. C.

[8] Vasita R., Katti D.S. (2006). Nanofibers and Their Applications in Tissue Engineering. Int. J. Nanomed..

[9] Hiwrale A., Bharati S., Pingale P., Rajput A. (2023). Nanofibers: A Current Era in Drug Delivery System. Heliyon.

[10] Zhong W., van Langenhove L. (2016). 3-Nanofibres for Medical Textiles. Advances in Smart Medical Textiles.

[11] Muppalaneni S. (2013). Polyvinyl Alcohol in Medicine and Pharmacy: A Perspective. J. Dev. Drugs.

[12] Kita M., Ogura Y., Honda Y., Hyon S.-H., Cha W.-I., Ikada Y. (1990). Evaluation of Polyvinyl Alcohol Hydrogel as a Soft Contact Lens Material. Graefes Arch. Clin. Exp. Ophthalmol..

[13] Khorasani M.T., Joorabloo A., Moghaddam A., Shamsi H., MansooriMoghadam Z. (2018). Incorporation of ZnO Nanoparticles into Heparinised Polyvinyl Alcohol/Chitosan Hydrogels for Wound Dressing Application. Int. J. Biol. Macromol..

[14] Teixeira M.A., Amorim M.T.P., Felgueiras H.P. (2020). Poly(Vinyl Alcohol)-Based Nanofibrous Electrospun Scaffolds for Tissue Engineering Applications. Polymers.

[15] Subramanian U.M., Vasanth K.S., Naveen N., Sivagnanam U.T. (2014). Fabrication of Polyvinyl Alcohol-Polyvinylpyrrolidone Blend Scaffolds via Electrospinning for Tissue Engineering Applications. Int. J. Polym. Mater. Polym. Biomater..

[16] Tampau A., González-Martínez C., Chiralt A. (2020). Polyvinyl Alcohol-Based Materials Encapsulating Carvacrol Obtained by Solvent Casting and Electrospinning. React. Funct. Polym..

[17] Cui Z., Zheng Z., Lin L., Si J., Wang Q., Peng X., Chen W. (2018). Electrospinning and Crosslinking of Polyvinyl Alcohol/Chitosan Composite Nanofiber for Transdermal Drug Delivery. Adv. Polym. Tech..

[18] Marega C., Maculan J., Rizzi G., Saini R., Cavaliere E., Gavioli L., Cattelan M., Giallongo G., Marigo A., Granozzi G. (2015). Polyvinyl Alcohol Electrospun Nanofibers Containing Ag Nanoparticles Used as Sensors for the Detection of Biogenic Amines. Nanotechnology.

[19] Zhang C., Yuan X., Wu L., Han Y., Sheng J. (2005). Study on Morphology of Electrospun Poly(Vinyl Alcohol) Mats. Eur. Polym. J..

[20] Buriti B., Figueiredo P.L.B., Passos M.F., da Silva J.K.R. (2024). Polymer-Based Wound Dressings Loaded with Essential Oil for the Treatment of Wounds: A Review. Pharmaceuticals.

[21] Liang X., Zhong H.J., Ding H., Yu B., Ma X., Liu X., Chong C.M., He J. (2024). Polyvinyl Alcohol (PVA)-Based Hydrogels: Recent Progress in Fabrication, Properties, and Multifunctional Applications. Polymers.

[22] Sa’adon S., Abd Razak S.I., Ismail A.E., Fakhruddin K. (2019). Drug-Loaded Poly-Vinyl Alcohol Electrospun Nanofibers for Transdermal Drug Delivery: Review on Factors Affecting the Drug Release. Procedia Comput. Sci..

[23] Zahra F.T., Quick Q., Mu R. (2023). Electrospun PVA Fibers for Drug Delivery: A Review. Polymers.

[24] Tahalyani J., Akhtar M.J., Kar K.K. (2021). Heterolayered Composite of Carbon Nanofibers Sandwiched between Poly(Ethylene Terephthalate) and Polyurethane for Flexible Electromagnetic Shielding Application. ACS Appl. Nano Mater..

[25] Bubakir M., Li H., Barhoum A., Yang W., Barhoum A., Hamdy A., Makhlouf M.B. (2017). Advances in Melt Electrospinning Technique. Handbook of Nanofibers.

[26] Aziz S., Heidarian P., Mathel V., McNally T., Peijs T., Nanjundan A.K., Varley R.J., Halley P.J., Vandi L.-J. (2025). Bioplastic Fibres: Processing, Properties, Applications and Challenges. Adv. Fiber Mater..

[27] Vu T.H.N., Morozkina S.N., Uspenskaya M. (2022). V Study of the Nanofibers Fabrication Conditions from the Mixture of Poly(Vinyl Alcohol) and Chitosan by Electrospinning Method. Polymers.

[28] Fathollahipour S., Abouei Mehrizi A., Ghaee A., Koosha M. (2015). Electrospinning of PVA/Chitosan Nanocomposite Nanofibers Containing Gelatin Nanoparticles as a Dual Drug Delivery System. J. Biomed. Mater. Res. A.

[29] Navaei F., Zandi M., Ganjloo A., Dardmeh N. (2025). Fabrication of Magnetic Nanoparticle-Enhanced Bi-Layer Films: Fe_3_O_4_-Loaded Electrospun PVA/Gelatin Nanofibers on a Balangu Seed Mucilage-Gelatin Base. Ind. Crops Prod..

[30] Natarelli C.V.L., de Barros H.E.A., Freitas H.R., Bufalo T.C.E., Dias E.S., de Oliveira J.E., Marconcini J.M. (2023). PVA/Zein Nanofibers Obtained by Solution Blow Spinning. J. Mater. Sci..

[31] Ullah S., Hashmi M., Shi J., Kim I.S. (2023). Fabrication of Electrospun PVA/Zein/Gelatin Based Active Packaging for Quality Maintenance of Different Food Items. Polymers.

[32] Salim S.A., Loutfy S.A., El-Fakharany E.M., Taha T.H., Hussien Y., Kamoun E.A. (2021). Influence of Chitosan and Hydroxyapatite Incorporation on Properties of Electrospun PVA/HA Nanofibrous Mats for Bone Tissue Regeneration: Nanofibers Optimization and in-Vitro Assessment. J. Drug Deliv. Sci. Technol..

[33] Shalumon K.T., Anulekha K.H., Nair S.V., Nair S.V., Chennazhi K.P., Jayakumar R. (2011). Sodium Alginate/Poly(Vinyl Alcohol)/Nano ZnO Composite Nanofibers for Antibacterial Wound Dressings. Int. J. Biol. Macromol..

[34] Norouzi F., Pourmadadi M., Yazdian F., Khoshmaram K., Mohammadnejad J., Sanati M.H., Chogan F., Rahdar A., Baino F. (2022). PVA-Based Nanofibers Containing Chitosan Modified with Graphene Oxide and Carbon Quantum Dot-Doped TiO2 Enhance Wound Healing in a Rat Model. J. Funct. Biomater..

[35] Zhai H., Liu J., Liu Z., Li Y. (2025). Functional Graphene Fiber Materials for Advanced Wearable Applications. Adv. Fiber Mater..

[36] Medeiros E.S., Glenn G.M., Klamczynski A.P., Orts W.J., Mattoso L.H.C. (2009). Solution Blow Spinning: A New Method to Produce Micro- and Nanofibers from Polymer Solutions. J. Appl. Polym. Sci..

[37] Xue J., Wu T., Dai Y., Xia Y. (2019). Electrospinning and Electrospun Nanofibers: Methods, Materials, and Applications. Chem. Rev..

[38] Liu L., Chang D., Gao C. (2024). A Review of Multifunctional Nanocomposite Fibers: Design, Preparation and Applications. Adv. Fiber Mater..

[39] Rogalski J.J., Bastiaansen C.W.M., Peijs T. (2017). Rotary Jet Spinning Review—A Potential High Yield Future for Polymer Nanofibers. Nanocomposites.

[40] Song J., Lin X., Ee L.Y., Li S.F.Y., Huang M. (2023). A Review on Electrospinning as Versatile Supports for Diverse Nanofibers and Their Applications in Environmental Sensing. Adv. Fiber Mater..

[41] Li C., Qiu M., Li R., Li X., Wang M., He J., Lin G., Xiao L., Qian Q., Chen Q. (2022). Electrospinning Engineering Enables High-Performance Sodium-Ion Batteries. Adv. Fiber Mater..

[42] Mirjalili M., Zohoori S. (2016). Review for Application of Electrospinning and Electrospun Nanofibers Technology in Textile Industry. J. Nanostructure Chem..

[43] Keirouz A., Wang Z., Reddy V.S., Nagy Z.K., Vass P., Buzgo M., Ramakrishna S., Radacsi N. (2023). The History of Electrospinning: Past, Present, and Future Developments. Adv. Mater. Technol..

[44] Gao Y., Zhang J., Su Y., Wang H., Wang X.-X., Huang L.-P., Yu M., Ramakrishna S., Long Y.-Z. (2021). Recent Progress and Challenges in Solution Blow Spinning. Mater. Horiz..

[45] Mobaraki M., Liu M., Masoud A.-R., Mills D.K. (2023). Biomedical Applications of Blow-Spun Coatings, Mats, and Scaffolds—A Mini-Review. J. Compos. Sci..

[46] Oliveira J.E., Mattoso L.H.C., Orts W.J., Medeiros E.S. (2013). Structural and Morphological Characterization of Micro and Nanofibers Produced by Electrospinning and Solution Blow Spinning: A Comparative Study. Adv. Mater. Sci. Eng..

[47] Ellison C.J., Phatak A., Giles D.W., Macosko C.W., Bates F.S. (2007). Melt Blown Nanofibers: Fiber Diameter Distributions and Onset of Fiber Breakup. Polymer.

[48] da Silva Parize D.D., Foschini M.M., de Oliveira J.E., Klamczynski A.P., Glenn G.M., Marconcini J.M., Mattoso L.H.C. (2016). Solution Blow Spinning: Parameters Optimization and Effects on the Properties of Nanofibers from Poly(Lactic Acid)/Dimethyl Carbonate Solutions. J. Mater. Sci..

[49] González-Benito J., Zuñiga-Prado S., Najera J., Olmos D. (2024). Non-Woven Fibrous Polylactic Acid/Hydroxyapatite Nanocomposites Obtained via Solution Blow Spinning: Morphology, Thermal and Mechanical Behavior. Nanomaterials.

[50] Oliveira J.E., Moraes E.A., Costa R.G.F., Afonso A.S., Mattoso L.H.C., Orts W.J., Medeiros E.S. (2011). Nano and Submicrometric Fibers of Poly(D,L-Lactide) Obtained by Solution Blow Spinning: Process and Solution Variables. J. Appl. Polym. Sci..

[51] Nikolić N., Olmos D., González-Benito J. (2024). Key Advances in Solution Blow Spinning of Polylactic-Acid-Based Materials: A Prospective Study on Uses and Future Applications. Polymers.

[52] Yu J.H., Fridrikh S.V., Rutledge G.C. (2006). The Role of Elasticity in the Formation of Electrospun Fibers. Polymer.

[53] Yu H., Tan Z. (2025). Electrospinning Parameters. Introduction to Electrospinning and Nanofiber.

[54] Palangetic L., Reddy N.K., Srinivasan S., Cohen R.E., McKinley G.H., Clasen C. (2014). Dispersity and Spinnability: Why Highly Polydisperse Polymer Solutions Are Desirable for Electrospinning. Polymer.

[55] Souza R.J., Soares Filho J.E., Simões T.A., Oliveira J.E., Medeiros E.S. (2024). Experimental Investigation of Solution Blow Spinning Nozzle Geometry and Processing Parameters on Fiber Morphology. ACS Appl. Polym. Mater..

[56] Matabola K.P., Moutloali R.M. (2013). The Influence of Electrospinning Parameters on the Morphology and Diameter of Poly(Vinyledene Fluoride) Nanofibers- Effect of Sodium Chloride. J. Mater. Sci..

[57] Nikolić N., Olmos D., Kramar A., González-Benito J. (2024). Effect of Collector Rotational Speed on the Morphology and Structure of Solution Blow Spun Polylactic Acid (PLA). Polymers.

[58] González-Benito J., Lorente M.A., Olmos D., Kramar A. (2023). Solution Blow Spinning to Prepare Preferred Oriented Poly(Ethylene Oxide) Submicrometric Fibers. Fibers.

[59] Yang G., Liao D., Chen A., Li C., Bashir M.S. (2024). Preparation of Poly(Vinyl Alcohol) with Enhanced Stereoselectivity via Hydrolysis of Poly(Vinyl Acetate) Produced by Radical Polymerization Using 7-Membered Ring Controlling Agents. J. Mol. Struct..

[60] Zhong Y., Lin Q., Yu H., Shao L., Cui X., Pang Q., Zhu Y., Hou R. (2024). Construction Methods and Biomedical Applications of PVA-Based Hydrogels. Front. Chem..

[61] Nagara Y., Nakano T., Okamoto Y., Gotoh Y., Nagura M. (2001). Properties of Highly Syndiotactic Poly(Vinyl Alcohol). Polymer.

[62] Cao J., Zhao X., Ye L. (2022). Super-Strong and Anti-Tearing Poly(Vinyl Alcohol)/Graphene Oxide Nano-Composite Hydrogels Fabricated by Formation of Multiple Crosslinking Bonding Network Structure. J. Ind. Eng. Chem..

[63] de Barros H.E.A., Natarelli C.V.L., Santos I.A., Soares L.S., Nunes Carvalho E.E., de Oliveira J.E., Franco M., Vilas Boas E.V.d.B. (2024). Development of Poly(Vinyl Alcohol) Nanofibers Incorporated with Aqueous Plant Extracts by Solution Blow Spinning and Their Application as Strawberry Coatings. J. Food Eng..

[64] Vu N.Q., Le T.M., Ngo A.N.B., Nguyen M.H.T., Liao Y.-C., Tran T.T. (2026). Engineering Functional PVA: A Comprehensive Review of Chemical Modifications and Prospective Developments. ACS Polym. AU.

[65] Razumova L.L., Veretennikova A.A., Zaikov G.Y., Vol’f L.A. (1983). Degradation of Polyvinyl Alcohol Surgical Thread. Polym. Sci. USSR.

[66] Adelnia H., Ensandoost R., Shebbrin Moonshi S., Gavgani J.N., Vasafi E.I., Ta H.T. (2022). Freeze/Thawed Polyvinyl Alcohol Hydrogels: Present, Past and Future. Eur. Polym. J..

[67] Zhao Y., Yang X., Zhang Q., Liang N., Xiang Y., Qin M. (2020). Crack Resistance and Mechanical Properties of Polyvinyl Alcohol Fiber-Reinforced Cement-Stabilized Macadam Base. J. Adv. Civ. Eng..

[68] Hu F., Lu H., Xu G., Lv L., Chen L., Shao Z. (2022). Carbon Quantum Dots Improve the Mechanical Behavior of Polyvinyl Alcohol/Polyethylene Glycol Hydrogel. J. Appl. Polym. Sci..

[69] Baker M.I., Walsh S.P., Schwartz Z., Boyan B.D. (2012). A Review of Polyvinyl Alcohol and Its Uses in Cartilage and Orthopedic Applications. J. BioMed. Mater. Res. B Appl. Biomater..

[70] Saini I., Sharma A., Dhiman R., Aggarwal S., Ram S., Sharma P. (2017). Grafted SiC Nanocrystals: For Enhanced Optical, Electrical and Mechanical Properties of Polyvinyl Alcohol. J. Alloys Compd..

[71] Ye Z., Lu H., Jia E., Chen J., Fu L. (2022). Organic Solvents Enhance Polyvinyl Alcohol/Polyethylene Glycol Self-Healing Hydrogels for Artificial Cartilage. Polym. Adv. Technol..

[72] Xiong J., Chen S., Choi Y., Matsugi K. (2021). Development of Polyvinyl Alcohol-Based Carbon Nano Fiber Sheet for Thermal Interface Material. Sci. Rep..

[73] Liu F., Ni Q.Q., Murakami Y. (2013). Preparation of Magnetic Polyvinyl Alcohol Composite Nanofibers with Homogenously Dispersed Nanoparticles and High Water Resistance. Text. Res. J..

[74] Liu S., Li D., Wang Y., Zhou G., Ge K., Jiang L. (2023). Adhesive, Antibacterial and Double Crosslinked Carboxylated Polyvinyl Alcohol/Chitosan Hydrogel to Enhance Dynamic Skin Wound Healing. Int. J. Biol. Macromol..

[75] Khorasani M.T., Joorabloo A., Adeli H., Mansoori-Moghadam Z., Moghaddam A. (2019). Design and Optimization of Process Parameters of Polyvinyl (Alcohol)/Chitosan/Nano Zinc Oxide Hydrogels as Wound Healing Materials. Carbohydr. Polym..

[76] Fatema U.K., Rahman M.M., Islam M.R., Mollah M.Y.A., Susan M.A.B.H. (2018). Silver/Poly(Vinyl Alcohol) Nanocomposite Film Prepared Using Water in Oil Microemulsion for Antibacterial Applications. J. Colloid Interface Sci..

[77] Kadhim K., Agool I., Hashim A. (2016). Synthesis of (PVA-PEG-PVP-TiO2) Nanocomposites for Antibacterial Application. Mater. Focus.

[78] Merkle V.M., Ammann K.R., DeCook J.K., Tran P.L., Slepian M.-J., Wu X. In Vitro Biocompatibility of Coaxial Electrospun Scaffolds for Cardiovascular Tissue Engineering. Proceedings of the ASME 2013 Summer Bioengineering Conference.

[79] Aytimur A., Uslu I. (2014). Promising Materials for Wound Dressing: PVA/PAA/PVP Electrospun Nanofibers. Polym. Plast. Technol. Eng..

[80] Renkler N.Z., Babar Z.U.D., Barra M., Cruz-Maya I., De Santis R., di Girolamo R., Marelli M., Ferretti A.M., Zaheer A., Iannotti V. (2025). Optimizing Electrospun PVA Fibers with MXene Integration for Biomedical Applications. Macromol. Mater. Eng..

[81] Türkoğlu G.C., Khomarloo N., Mohsenzadeh E., Gospodinova D.N., Neznakomova M., Salaün F. (2024). PVA-Based Electrospun Materials—A Promising Route to Designing Nanofiber Mats with Desired Morphological Shape—A Review. Int. J. Mol. Sci..

[82] Kamoun E.A., Loutfy S.A., Hussein Y., Kenawy E.-R.S. (2021). Recent Advances in PVA-Polysaccharide Based Hydrogels and Electrospun Nanofibers in Biomedical Applications: A Review. Int. J. Biol. Macromol..

[83] Oliveira A.S., Silva J.C., Loureiro M.V., Marques A.C., Kotov N.A., Colaço R., Serro A.P. (2023). Super-Strong Hydrogel Composites Reinforced with PBO Nanofibers for Cartilage Replacement. Macromol. Biosci..

[84] Yang F., Zhao J., Koshut W.J., Watt J., Riboh J.C., Gall K., Wiley B.J. (2020). A Synthetic Hydrogel Composite with the Mechanical Behavior and Durability of Cartilage. Adv. Funct. Mater..

[85] Liu J., Lin S., Liu X., Qin Z., Yang Y., Zang J., Zhao X. (2020). Fatigue-Resistant Adhesion of Hydrogels. Nat. Commun..

[86] Liu H., Du C., Liao L., Zhang H., Zhou H., Zhou W., Ren T., Sun Z., Lu Y., Nie Z. (2022). Approaching Intrinsic Dynamics of MXenes Hybrid Hydrogel for 3D Printed Multimodal Intelligent Devices with Ultrahigh Superelasticity and Temperature Sensitivity. Nat. Commun..

[87] Lu L., Huang Z., Li X., Li X., Cui B., Yuan C., Guo L., Liu P., Dai Q. (2022). A High-Conductive, Anti-Freezing, Antibacterial and Anti-Swelling Starch-Based Physical Hydrogel for Multifunctional Flexible Wearable Sensors. Int. J. Biol. Macromol..

[88] Cao J., He G., Ning X., Chen X., Fan L., Yang M., Yin Y., Cai W. (2022). Preparation and Properties of O-Chitosan Quaternary Ammonium Salt/Polyvinyl Alcohol/Graphene Oxide Dual Self-Healing Hydrogel. Carbohydr. Polym..

[89] Liu X., Yang K., Chang M., Wang X., Ren J. (2020). Fabrication of Cellulose Nanocrystal Reinforced Nanocomposite Hydrogel with Self-Healing Properties. Carbohydr. Polym..

[90] Su Y., Zhang X., Wei Y., Gu Y., Xu H., Liao Z., Zhao L., Du J., Hu Y., Lian X. (2023). Nanocatalytic Hydrogel with Rapid Photodisinfection and Robust Adhesion for Fortified Cutaneous Regeneration. ACS Appl. Mater. Interfaces.

[91] Escobar C., Figueroa T., González L., Ruíz I., Aguayo C.R., Toledo J.R., Garrido Á.R.R., Larenas-Muñoz F., Fernández K. (2026). Poly(Vinyl Alcohol) (PVA)/Graphene Oxide (GO)/Vitamin A Palmitate (VAP) Hydrogels for Wound Care: Integrating Mechanical Robustness, Photoprotection, and Enhanced Bioactivity. ACS Appl. Polym. Mater..

[92] Chen S., Miyazaki T., Itoh M., Matsumoto H., Moro-Oka Y., Tanaka M., Miyahara Y., Suganami T., Matsumoto A. (2022). A Porous Reservoir-Backed Boronate Gel Microneedle for Efficient Skin Penetration and Sustained Glucose-Responsive Insulin Delivery. Gels.

[93] Thakur S., Anjum M.M., Jaiswal S., Kumar A., Deepak P., Anand S., Singh S., Rajinikanth P.S. (2023). Novel Synergistic Approach: Tazarotene-Calcipotriol-Loaded-PVA/PVP-Nanofibers Incorporated in Hydrogel Film for Management and Treatment of Psoriasis. Mol. Pharm..

[94] Li J., Li H., Wu C., Zhang W. (2022). PVA-AAm-AG Multi-Network Hydrogel with High Mechanical Strength and Cell Adhesion. Polymer.

[95] Branco A.C., Oliveira A.S., Monteiro I., Nolasco P., Silva D.C., Figueiredo-Pina C.G., Colaço R., Serro A.P. (2022). PVA-Based Hydrogels Loaded with Diclofenac for Cartilage Replacement. Gels.

[96] Abouzeid R.E., Khiari R., Salama A., Diab M., Beneventi D., Dufresne A. (2020). In Situ Mineralization of Nano-Hydroxyapatite on Bifunctional Cellulose Nanofiber/Polyvinyl Alcohol/Sodium Alginate Hydrogel Using 3D Printing. Int. J. Biol. Macromol..

[97] Hou R., Nie L., Du G., Xiong X., Fu J. (2015). Natural Polysaccharides Promote Chondrocyte Adhesion and Proliferation on Magnetic Nanoparticle/PVA Composite Hydrogels. Colloids Surf. B Biointerfaces.

[98] Nie L., Li X., Chang P., Liu S., Wei Q., Guo Q., Wu Q., Fan L., Okoro O.V., Shavandi A. (2022). A Fast Method for in Vitro Biomineralization of PVA/Alginate/Biphasic Calcium Phosphate Hydrogel. Mater. Lett..

[99] Qin Y., Mo J., Liu Y., Zhang S., Wang J., Fu Q., Wang S., Nie S. (2022). Stretchable Triboelectric Self-powered Sweat Sensor Fabricated from Self-healing Nanocellulose Hydrogels. Adv. Funct. Mater..

[100] Fan L., Xie J., Zheng Y., Wei D., Yao D., Zhang J., Zhang T. (2020). Antibacterial, Self-Adhesive, Recyclable, and Tough Conductive Composite Hydrogels for Ultrasensitive Strain Sensing. ACS Appl. Mater. Interfaces.

[101] Pan X., Wang Q., He P., Liu K., Ni Y., Chen L., Ouyang X., Huang L., Wang H., Xu S. (2020). A Bionic Tactile Plastic Hydrogel-Based Electronic Skin Constructed by a Nerve-like Nanonetwork Combining Stretchable, Compliant, and Self-Healing Properties. Chem. Eng. J..

[102] Song M., Yu H., Zhu J., Ouyang Z., Abdalkarim S.Y.H., Tam K.C., Li Y. (2020). Constructing Stimuli-Free Self-Healing, Robust and Ultrasensitive Biocompatible Hydrogel Sensors with Conductive Cellulose Nanocrystals. Chem. Eng. J..

[103] Li L., Zhang Y., Lu H., Wang Y., Xu J., Zhu J., Zhang C., Liu T. (2020). Cryopolymerization Enables Anisotropic Polyaniline Hybrid Hydrogels with Superelasticity and Highly Deformation-Tolerant Electrochemical Energy Storage. Nat. Commun..

[104] Zhou Y., Wan C., Yang Y., Yang H., Wang S., Dai Z., Ji K., Jiang H., Chen X., Long Y. (2019). Highly Stretchable, Elastic, and Ionic Conductive Hydrogel for Artificial Soft Electronics. Adv. Funct. Mater..

[105] Li W., Li X., Zhang X., Wu J., Tian X., Zeng M.-J., Qu J., Yu Z.-Z. (2020). Flexible Poly (Vinyl Alcohol)–Polyaniline Hydrogel Film with Vertically Aligned Channels for an Integrated and Self-Healable Supercapacitor. ACS Appl. Energy Mater..

[106] Dai X., Long Y., Jiang B., Guo W., Sha W., Wang J., Cong Z., Chen J., Wang B., Hu W. (2022). Ultra-Antifreeze, Ultra-Stretchable, Transparent, and Conductive Hydrogel for Multi-Functional Flexible Electronics as Strain Sensor and Triboelectric Nanogenerator. Nano Res..

[107] Luo X., Zhu L., Wang Y., Li J., Nie J., Wang Z.L. (2021). A Flexible Multifunctional Triboelectric Nanogenerator Based on MXene/PVA Hydrogel. Adv. Funct. Mater..

[108] Hu K., He P., Zhao Z., Huang L., Liu K., Lin S., Zhang M., Wu H., Chen L., Ni Y. (2021). Nature-Inspired Self-Powered Cellulose Nanofibrils Hydrogels with High Sensitivity and Mechanical Adaptability. Carbohydr. Polym..

[109] Han L., Zhou Q., Chen D., Qu R., Liu L., Chen Y., Yang J., Song X., Physicochemical S.A., Aspects E. (2022). Flexible Sensitive Hydrogel Sensor with Self-Powered Capability. Colloids Surf. A Physicochem. Eng. Asp..

[110] Chen X. (2017). Making Electrodes Stretchable. Small Methods.

[111] Qu J., Zhao X., Liang Y., Zhang T., Ma P.X., Guo B. (2018). Antibacterial Adhesive Injectable Hydrogels with Rapid Self-Healing, Extensibility and Compressibility as Wound Dressing for Joints Skin Wound Healing. Biomaterials.

[112] Montaser A.S., Rehan M., El-Naggar M. (2019). PH-Thermosensitive Hydrogel Based on Polyvinyl Alcohol/Sodium Alginate/N-Isopropyl Acrylamide Composite for Treating Re-Infected Wounds. Int. J. Biol. Macromol..

[113] Yang J., Cao Z. (2017). Glucose-Responsive Insulin Release: Analysis of Mechanisms, Formulations, and Evaluation Criteria. J. Control Release.

[114] Shamloo A., Aghababaie Z., Afjoul H., Jami M., Bidgoli M.R., Vossoughi M., Ramazani A., Kamyabhesari K. (2021). Fabrication and Evaluation of Chitosan/Gelatin/PVA Hydrogel Incorporating Honey for Wound Healing Applications: An in Vitro, in Vivo Study. Int. J. Pharm..

[115] Abbasi A.R., Sohail M., Minhas M.U., Khaliq T., Kousar M., Khan S., Hussain Z., Munir A. (2020). Bioinspired Sodium Alginate Based Thermosensitive Hydrogel Membranes for Accelerated Wound Healing. Int. J. Biol. Macromol..

[116] Eivazzadeh-Keihan R., Khalili F., Aliabadi H.A.M., Maleki A., Madanchi H., Ziabari E.Z., Bani M.S. (2020). Alginate Hydrogel-Polyvinyl Alcohol/Silk Fibroin/Magnesium Hydroxide Nanorods: A Novel Scaffold with Biological and Antibacterial Activity and Improved Mechanical Properties. Int. J. Biol. Macromol..

[117] Liu B., Fu R., Duan Z., Zhu C., Deng J., Fan D. (2022). Ionic Liquid-Based Non-Releasing Antibacterial, Anti-Inflammatory, High-Transparency Hydrogel Coupled with Electrical Stimulation for Infected Diabetic Wound Healing. Compos. B Eng..

[118] Wang Z., Sheng K., Zhou Z., Li H., Zhang Z., Xiong M., Huang J. (2023). High Ionic Conductivity of “Room Temperature Molten Salt” Based PVA Hydrogel Electrolyte Prepared by Electron Beam Irradiation. Radiat. Phys. Chem..

[119] Yao J., Hui J., Yang J., Yao J., Hu C., Fan D. (2022). Sprayable Nanodrug-Loaded Hydrogels with Enzyme-Catalyzed Semi-Inter Penetrating Polymer Network (Semi-IPN) for Solar Dermatitis. Nano Res..

[120] Huang X., Xu L., Yu X., Li Y., Huang Z., Xu R., Zeng W., Zhang Z., Li W., Deng F. (2023). Near-Infrared Light-Responsive Multifunctional Hydrogel Releasing Peptide-Functionalized Gold Nanorods Sequentially for Diabetic Wound Healing. J. Colloid Interface Sci..

[121] Qiao B., Pang Q., Yuan P., Luo Y., Ma L. (2020). Smart Wound Dressing for Infection Monitoring and NIR-Triggered Antibacterial Treatment. Biomater. Sci..

[122] Chelu M., Popa M., Calderon Moreno J.M. (2025). Applications of Hydrogels in Emergency Therapy. Gels.

[123] Fuchs S., Shariati K., Ma M. (2020). Specialty Tough Hydrogels and Their Biomedical Applications. Adv. Healthc. Mater..

[124] Abdulhussain R., Adebisi A., Conway B.R., Asare-Addo K. (2023). Electrospun Nanofibers: Exploring Process Parameters, Polymer Selection, and Recent Applications in Pharmaceuticals and Drug Delivery. J. Drug Deliv. Sci. Technol..

[125] Homer W.J.A., Lisnenko M., Hauzerova S., Heczkova B., Gardner A.C., Kostakova E.K., Topham P.D., Jencova V., Theodosiou E. (2024). Thermally Stabilised Poly(Vinyl Alcohol) Nanofibrous Materials Produced by Scalable Electrospinning: Applications in Tissue Engineering. Polymers.

[126] Nataraj D., Reddy R., Reddy N. (2020). Crosslinking Electrospun Poly (Vinyl) Alcohol Fibers with Citric Acid to Impart Aqueous Stability for Medical Applications. Eur. Polym. J..

[127] Ghajarieh A., Habibi S., Talebian A. (2021). Biomedical Applications of Nanofibers. Russ. J. Appl. Chem..

[128] Zeng J., Aigner A., Czubayko F., Kissel T., Wendorff J.H., Greiner A. (2005). Poly(Vinyl Alcohol) Nanofibers by Electrospinning as a Protein Delivery System and the Retardation of Enzyme Release by Additional Polymer Coatings. Biomacromolecules.

[129] de Castro K.C., Burga-Sánchez J., Nogueira Campos M.G., Mei L.H.I. (2020). Water-Based Synthesis of Photocrosslinked Hyaluronic Acid/Polyvinyl Alcohol Membranes via Electrospinning. RSC Adv..

[130] Chouhan D., Jain D., Singh A.K., Jha A., Londhe R.D., Mishra B., Tiwari V. (2026). Electrospun Hyaluronic Acid/Polyvinyl Alcohol Nanofibers Encapsulating Defactinib as Bioactive Dressings for Burn Wound Therapy. ACS Appl. Bio Mater..

[131] Wang C., Yan K.-W., Lin Y.-D., Hsieh P.C.H. (2010). Biodegradable Core/Shell Fibers by Coaxial Electrospinning: Processing, Fiber Characterization, and Its Application in Sustained Drug Release. Macromolecules.

[132] Alharbi H.F., Luqman M., Khalil K.A., Elnakady Y.A., Abd-Elkader O.H., Rady A.M., Alharthi N.H., Karim M.R. (2018). Fabrication of Core-Shell Structured Nanofibers of Poly (Lactic Acid) and Poly (Vinyl Alcohol) by Coaxial Electrospinning for Tissue Engineering. Eur. Polym. J..

[133] Lin H.-Y., Hsin-Hung C., Shih-Hsin C., Ni T.-S. (2013). Pectin-Chitosan-PVA Nanofibrous Scaffold Made by Electrospinning and Its Potential Use as a Skin Tissue Scaffold. J. Biomater. Sci. Polym. Ed..

[134] Asran A.S., Razghandi K., Aggarwal N., Michler G.H., Groth T. (2010). Nanofibers from Blends of Polyvinyl Alcohol and Polyhydroxy Butyrate As Potential Scaffold Material for Tissue Engineering of Skin. Biomacromolecules.

[135] Alhosseini S., Moztarzadeh F., Mozafari M., Asgari S., Dodel M., Samadikuchaksaraei A., Kargozar S., Jalali N. (2012). Synthesis and Characterization of Electrospun Polyvinyl Alcohol Nanofibrous Scaffolds Modified by Blending with Chitosan for Neural Tissue Engineering. Int. J. Nanomed..

[136] Ma Y., Deng B., He R., Huang P. (2024). Advancements of 3D Bioprinting in Regenerative Medicine: Exploring Cell Sources for Organ Fabrication. Heliyon.

[137] Zhou Y., Yang H., Liu X., Mao J., Gu S., Xu W. (2013). Electrospinning of Carboxyethyl Chitosan/Poly (Vinyl Alcohol)/Silk Fibroin Nanoparticles for Wound Dressings. Int. J. Biol. Macromol..

[138] Liu Y.Y., Yu H.C., Liu Y., Liang G., Zhang T., Hu Q.X. (2016). Dual Drug Spatiotemporal Release from Functional Gradient Scaffolds Prepared Using 3D Bioprinting and Electrospinning. Polym. Eng. Sci..

[139] Askari P., Zahedi P., Rezaeian I. (2016). Three-Layered Electrospun PVA/PCL/PVA Nanofibrous Mats Containing Tetracycline Hydrochloride and Phenytoin Sodium: A Case Study on Sustained Control Release, Antibacterial, and Cell Culture Properties. J. Appl. Polym. Sci..

[140] Fathi-Azarbayjani A., Qun L., Chan Y.W., Chan S.Y. (2010). Novel Vitamin and Gold-Loaded Nanofiber Facial Mask for Topical Delivery. AAPS PharmSciTech.

[141] Miyamoto A., Lee S., Cooray N.F., Lee S., Mori M., Matsuhisa N., Jin H., Yoda L., Yokota T., Itoh A. (2017). Inflammation-Free, Gas-Permeable, Lightweight, Stretchable on-Skin Electronics with Nanomeshes. Nat. Nanotechnol..

[142] Wang D., Zhang D., Li P., Yang Z., Mi Q., Yu L. (2021). Electrospinning of Flexible Poly(Vinyl Alcohol)/MXene Nanofiber-Based Humidity Sensor Self-Powered by Monolayer Molybdenum Diselenide Piezoelectric Nanogenerator. Nanomicro Lett..

[143] Nematollahi S., Maghsoudian S., Motasadizadeh H., Nouri Z., Azad K., Fatahi Y., Samadi N., Mahmoudieh M., Shaabani A., Dinarvand R. (2025). Polyhexamethylene Biguanidine Coated Silver Nanoparticles Embedded into Chitosan Thiourea/PVA Nanofibers as Wound Healing Mats: In Vitro and in Vivo Studies. Carbohydr. Polym..

[144] Sandra S., Anakha D.R., Silpa C., Vyshnavi T.V., Bhagiyalakshmi M., Yamuna R., Karthega M. (2024). Synthesis of Electrospun PVA/Chitosan Nanofibrous Scaffold Impregnated with CuO Nanoparticles for Wound Healing. Cellulose.

[145] Alavarse A.C., de Oliveira Silva F.W., Colque J.T., da Silva V.M., Prieto T., Venancio E.C., Bonvent J.J. (2017). Tetracycline Hydrochloride-Loaded Electrospun Nanofibers Mats Based on PVA and Chitosan for Wound Dressing. Mater. Sci. Eng. C.

[146] Ahmed R., Tariq M., Ali I., Asghar R., Noorunnisa Khanam P., Augustine R., Hasan A. (2018). Novel Electrospun Chitosan/Polyvinyl Alcohol/Zinc Oxide Nanofibrous Mats with Antibacterial and Antioxidant Properties for Diabetic Wound Healing. Int. J. Biol. Macromol..

[147] Cerqueira G.R.C., Gomes D.S., Victor R.S., Figueiredo L.R.F., Medeiros E.S., Neves G.A., Menezes R.R., Silva S.M.L. (2024). Development of PVA/Chitosan Nanofibers by a Green Route Using Solution Blow Spinning. J. Polym. Environ..

[148] Scaffaro R., Settanni L., Gulino E.F. (2024). Innovative Heat Assisted Solution Blow Spinning: PVA Mats with Enhanced Thermal Stabilization and Antimicrobial Efficacy. J. Text. Inst..

[149] Raimundo R.A., Silva V.D., Simões T.A., Medeiros E.S., Macedo D.A., Morales M.A. (2020). Ni/NiO-Carbon Composite Fibers Prepared by Solution Blow Spinning: Structure and Magnetic Properties. Ceram. Int..

[150] Snari R.M., Bayazeed A., Ibarhiam S.F., Alnoman R.B., Attar R., Abumelha H.M., El-Metwaly N.M. (2022). Solution Blowing Spinning of Polylactate/Polyvinyl Alcohol/ZnO Nanocomposite toward Green and Sustainable Preparation of Wound Dressing Nanofibrous Films. Microsc. Res. Tech..

[151] Tadele D.T., David D., Yim E., Mekonnen T.H. (2025). Development and Characterization of PVA-Zein/α-Tocopherol Nonwoven Mats for Functional Wound Dressing Applications. Colloids Surf. B Biointerfaces.

[152] Victor R.d.S., Gomes D.d.S., Santos A.M.d.C., Torres S.M., Neves G.d.A., Menezes R.R. (2024). 3D Nanofibrous Structures Formed of High Content Chitosan/PVA and Chitosan/PLA Blends Using Air-Heated Solution Blow Spinning (A-HSBS). J. Mater. Sci..

[153] Tan N.P.B., Paclijan S.S., Ali H.N.M., Hallazgo C.M.J.S., Lopez C.J.F., Ebora Y.C. (2019). Solution Blow Spinning (SBS) Nanofibers for Composite Air Filter Masks. ACS Appl. Nano Mater..

[154] Nikolić N., González-Benito J. (2025). Multifunctional Materials Constituted by Biodegradable PLA-Based Magnetic Submicrometric Fibers Prepared by Solution Blow Spinning. Mater. Today Chem..

[155] Bonan R.F., Bonan P.R.F., Batista A.U.D., Sampaio F.C., Albuquerque A.J.R., Moraes M.C.B., Mattoso L.H.C., Glenn G.M., Medeiros E.S., Oliveira J.E. (2015). In Vitro Antimicrobial Activity of Solution Blow Spun Poly(Lactic Acid)/Polyvinylpyrrolidone Nanofibers Loaded with Copaiba (*Copaifera* sp.) Oil. Mater. Sci. Eng. C.

[156] Santos A.M.C., Medeiros E.L.G., Blaker J.J., Medeiros E.S. (2016). Aqueous Solution Blow Spinning of Poly(Vinyl Alcohol) Micro- and Nanofibers. Mater. Lett..

[157] Medeiros E.L.G., Gomes D.S., Santos A.M.C., Vieira R.H., de Lima I.L., Rocha F.S., Castro-Filice L.d.S., Medeiros E.S., Neves G.A., Menezes R.R. (2021). 3D Nanofibrous Bioactive Glass Scaffolds Produced by One-Step Spinning Process. Ceram. Int..

[158] Pessoa A.L., Koblischka-Veneva A., Carvalho C.L., Zadorosny R., Koblischka M.R. (2021). Paramagnetic Meissner Effect and Current Flow in YBCO Nanofiber Mats. IEEE Trans. Appl. Supercond..

[159] Lyu H., Han Y., Zhang R., Wu Y., Wu X. (2022). Anti-Biofouling Multi-Modified Chitosan/Polyvinylalcohol Air-Blown Nanofibers for Selective Radionuclide Capture in Wastewater. Sep. Purif. Technol..

[160] Li Z., Cui Y., Zhou S., Wang S., Duan Y., Zhang J., Zong L. (2026). Scalable, Tough, and Nanohybrid Hydrogel Sub-Microfibers with 3D Hierarchical Structures for High Performance All-Day, Autonomous Water Collected Devices. J. Colloid Interface Sci..

[161] Fang G., Aghababaei F., Khan A., Riahi Z., Goksen G., Hadidi M., Yi G. (2026). Valorizing Animal Waste Protein through Spinning: Sustainable Nanofiber Innovation for Advanced Food Packaging. Food Qual. Saf..

[162] Asaduzzaman F. (2022). Enzyme Functionalized Solution Blown Nonwovens. Doctoral Dissertation.

[163] Tang C., Saquing C.D., Harding J.R., Khan S.A. (2010). In Situ Cross-Linking of Electrospun Poly(Vinyl Alcohol) Nanofibers. Macromolecules.

